# Structural elements promote architectural stripe formation and facilitate ultra-long-range gene regulation at a human disease locus

**DOI:** 10.1016/j.molcel.2023.03.009

**Published:** 2023-05-04

**Authors:** Liang-Fu Chen, Hannah Katherine Long, Minhee Park, Tomek Swigut, Alistair Nicol Boettiger, Joanna Wysocka

**Affiliations:** 1 Department of Chemical and Systems Biology, Stanford University School of Medicine, Stanford, CA 94305, USA; 2 Department of Developmental Biology, Stanford University School of Medicine, Stanford, CA 94305, USA; 3 Institute of Stem Cell Biology and Regenerative Medicine, Stanford University School of Medicine, Stanford, CA 94305, USA; 4 Howard Hughes Medical Institute, Stanford University School of Medicine, Stanford, CA 94305, USA

**Keywords:** enhancer, gene regulation, CTCF, loop extrusion, 3D genome architecture, stripe-associated structural element

## Abstract

Enhancer clusters overlapping disease-associated mutations in Pierre Robin sequence (PRS) patients regulate *SOX9* expression at genomic distances over 1.25 Mb. We applied optical reconstruction of chromatin architecture (ORCA) imaging to trace 3D locus topology during PRS-enhancer activation. We observed pronounced changes in locus topology between cell types. Subsequent analysis of single-chromatin fiber traces revealed that these ensemble-average differences arise through changes in the frequency of commonly sampled topologies. We further identified two CTCF-bound elements, internal to the *SOX9* topologically associating domain, which promote stripe formation, are positioned near the domain’s 3D geometric center, and bridge enhancer-promoter contacts in a series of chromatin loops. Ablation of these elements results in diminished *SOX9* expression and altered domain-wide contacts. Polymer models with uniform loading across the domain and frequent cohesin collisions recapitulate this multi-loop, centrally clustered geometry. Together, we provide mechanistic insights into architectural stripe formation and gene regulation over ultra-long genomic ranges.

## Highlights

• *SOX9* domain topology dynamically changes during a developmental transition
• Structural elements promote TAD-wide interactions, stripe formation, and transcription
• Structural elements are CTCF dependent and situated centrally in the 3D TAD structure
• Polymer simulations of the multi-loop model best recapitulate the topological features

## Graphical abstract

**Figure:**
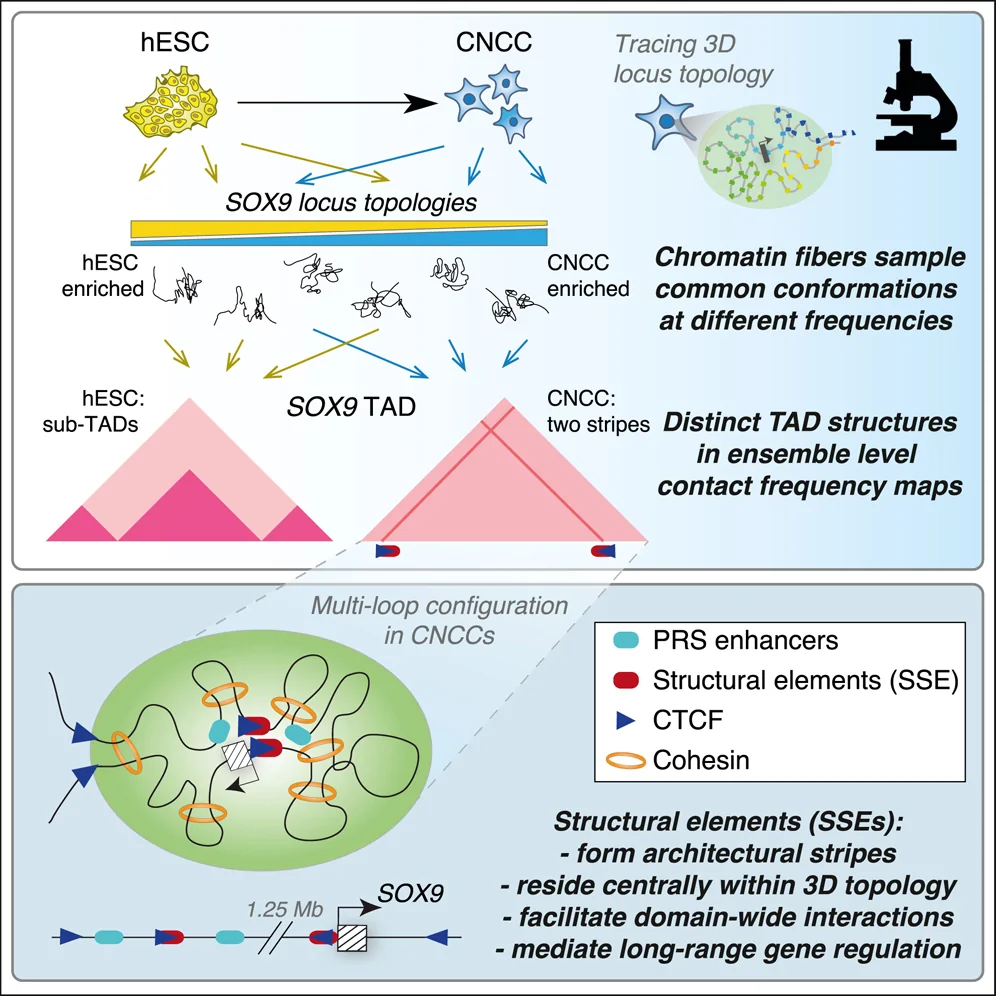

## Teaser

Enhancer clusters overlapping disease-associated mutations in Pierre Robin sequence (PRS) patients regulate *SOX9* expression at genomic distances over 1.25 Mb. Chen and Long et al. trace the 3D topology of the *SOX9* locus during PRS-enhancer activation and identify structural elements that promote domain-wide interactions and facilitate ultra-long-range gene regulation.

## Introduction

During development, gene expression is regulated in a dynamic and cell-type-specific manner. Much of this cell-type-specific gene regulation is mediated by the promoter-distal non-coding regulatory elements called enhancers.^1^^,^^2^ The mechanisms by which the enhancers control gene expression, especially across vast genomic distances, are an active area of inquiry, with much recent attention focused on the role of topologically associating domains (TADs). TADs were originally described from chromosome conformation capture-based studies as regions of self-interacting chromatin that are enriched for contacts within a domain and depleted for contacts between domains.^3^^,^^4^ Further fine-scale mapping of TADs has uncovered increasing details of the architecture within these domains, including contact points (also known as off-diagonal dots or loops), architectural stripes (also known as flares or lines), and sub-TADs.^5^^,^^6^ Recent single-cell studies revealed considerable structural heterogeneity of the chromatin organization at individual loci,^7^^,^^8^^,^^9^^,^^10^^,^^11^^,^^12^^,^^13^ indicating that TADs are dynamic structures that appear as an emergent property across a population of cells. Functionally, TADs can restrict the enhancer activity within a given locus,^14^^,^^15^ and disruption of TAD organization has been implicated in misregulation of gene expression and human disease.^16^^,^^17^^,^^18^^,^^19^

It has been proposed that TADs arise through the process of loop extrusion by the cohesin complex that actively spools DNA to create an extending DNA loop.^20^^,^^21^ TAD boundaries are frequently delimited by a genome-organizing factor, CCCTC-binding factor (CTCF), bound in a convergent orientation, which can stall and stabilize cohesin.^22^^,^^23^^,^^24^^,^^25^ Inducible degradation of cohesin or CTCF leads to the dissolution of TAD structures, although surprisingly, the impact on transcription is only moderate.^26^^,^^27^ In addition to its function at boundary elements, CTCF can be involved in the formation of regulatory neighborhoods within TADs^28^ or mediate enhancer-promoter contacts,^29^^,^^30^ suggesting a broad role for such sites in regulating transcription.

The *SOX9* locus provides a disease-relevant model for studying the long-range gene regulation and 3D genome organization. The *SOX9* gene—which encodes for a developmentally important transcription factor (TF)—is the only protein-coding gene immersed in an enormous gene desert within a two-megabase (Mb) TAD, one of the largest in the human genome. Given the broad developmental importance of SOX9, distinct non-coding mutations within the domain are associated with several human congenital disorders impacting the face, limbs, or sex determination.^31^^,^^32^^,^^33^^,^^34^^,^^35^ We previously characterized two ultra-long-range enhancer clusters (ECs) at the *SOX9* locus, 1.45 and 1.25 Mb upstream of *SOX9*, named as EC1.45 and EC1.25.^36^ These ECs overlap mutations identified for patients with an isolated craniofacial disorder called Pierre Robin Sequence (PRS)^31^^,^^37^ and regulate *SOX9* expression specifically in cranial neural crest cells (CNCCs), a transient embryonic cell type that forms the majority of craniofacial structures during development.^36^^,^^38^ Interestingly, active enhancer chromatin marks in CNCCs highlighted a third candidate regulatory element overlapping PRS patient deletions (termed E1.35). However, in contrast to the flanking EC1.45 and EC1.25 elements, E1.35 lacked autonomous enhancer activity in reporter assays, leaving its potential function in *SOX9* regulation unresolved. Furthermore, it remains unclear how EC1.45 and EC1.25 can achieve precise *SOX9* regulation across genomic distances of over 1.2 Mb—one of the longest-range functionally validated distal regulatory loci in the human genome. Precision in *SOX9* regulation is required to ensure normal craniofacial development, as even small 10%–15% changes in developmental *SOX9* dosage are sufficient to result in subtle but reproducible changes in lower jaw morphologies.^36^

Here, we use optical reconstruction of chromatin architecture (ORCA)^10^ to investigate the topological folding of the *SOX9* locus in two cellular states: (1) human embryonic stem cells (hESCs), where ECs are inactive and *SOX9* is lowly transcribed, and (2) CNCCs, where both PRS locus ECs and the *SOX9* gene are highly active. At the ensemble level, we observe sub-TAD structures and a paucity of long-range interactions in hESCs, whereas uniform TAD-wide interactions and the appearance of two architectural stripes are observed in CNCCs. Analysis of our ORCA data at the single-chromatin fiber level reveals that these differences arise through cell-type-specific changes in the frequency of topologies commonly sampled by both cell types. We further show that the two architectural stripes observed in CNCCs form at TAD-internal regions corresponding to, respectively, the E1.35 element and the *SOX9* promoter-proximal CTCF sites, which we refer to as stripe-associated structural elements (SSEs). These SSEs maintain a compact TAD structure in CNCCs and are centrally positioned within the 3D topology of the domain. This central positioning appears to facilitate TAD-wide contacts of the *SOX9* promoter, including multiway contacts with the PRS locus enhancers. Polymer simulations of loop extrusion show that the observed locus topologies and central 3D positioning of the structural elements are better approximated by a “multi-loop” model, compared with the previously proposed “reel-in” model of architectural stripe formation.^39^

## Results

### Pronounced changes in *SOX9* locus topology accompany neural crest cell differentiation

**Figure 1:**
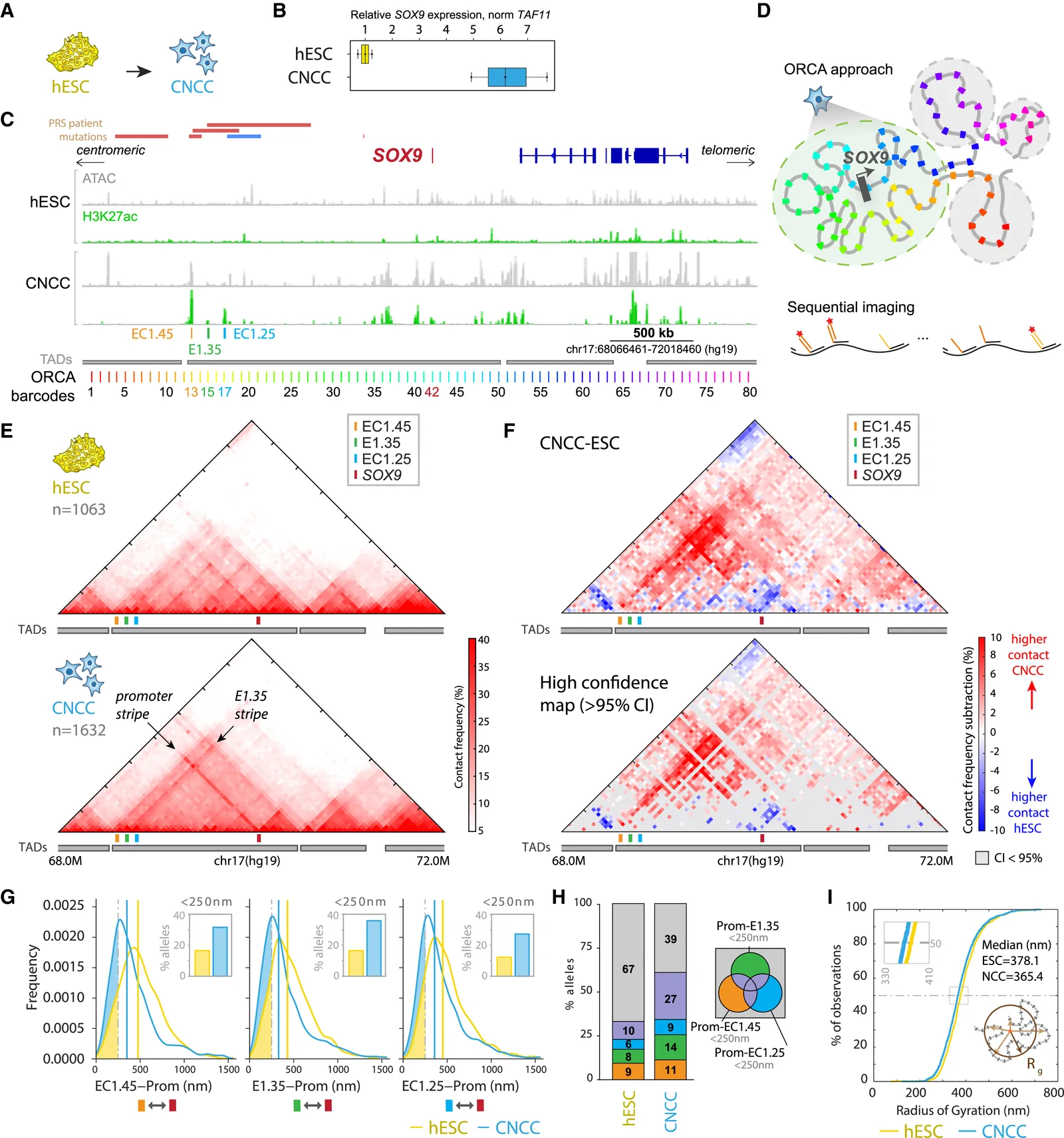
ORCA imaging reveals changes in domain topology during cranial neural crest cell differentiation (A) Schematic of hESC to CNCC differentiation. (B) Boxplot of *SOX9* expression measured by RT-ddPCR normalized to *TAF11* with hESC median set to 1. n = 3. (C) ATAC-seq and H3K27ac ChIP-seq data from hESCs and CNCCs for a 4 Mb locus flanking *SOX9*. Annotated features include enhancer clusters EC1.45 and EC1.25, element E1.35, PRS patient deletions (red) and translocation breakpoint cluster (blue),^31^^,^^42^^,^^43^ TADs (gray), and 80 ORCA barcodes (chr17:68066461-72018460, hg19). (D) Schematic of ORCA strategy. (E) ORCA contact frequency maps for pairwise distances < 250 nm for hESC (upper) and CNCCs (lower). Arrows: two domain-spanning stripes. (F) Contact frequency subtraction maps for CNCC-hESC (upper) and high-confidence map, confidence interval > 95% (lower). (G) Pairwise distance distributions for hESC (yellow) and CNCC (blue) for EC1.45, E1.35, and EC1.25 to the *SOX9* promoter (left to right). Inset: percentage of traces with pairwise distances < 250 nm. (H) Percentage of traces with promoter proximity (<250 nm) to EC1.45, E1.35, and/or EC1.25 including multiway interactions for hESCs and CNCCs. (I) The cumulative distribution plot for the *SOX9* TAD radius of gyration, hESC (yellow) and CNCCs (blue). See also Figure S1 and Table S1 and S2.

During *in vitro* differentiation of hESC to CNCCs, *SOX9* expression is upregulated around 6-fold (Figures 1A and B), and multiple enhancers are activated within the *SOX9* TAD, including the two PRS locus ECs EC1.45 and EC1.25 (Figures 1C and S1A).^36^ We used ORCA, a multiplexed sequential fluorescence *in situ* hybridization (FISH) methodology, to image ∼4 Mb of chromatin encompassing the *SOX9* regulatory domain and flanking TADs in hESCs and CNCCs (Figure 1D).^10^ To examine the population-average topology from hundreds of individual chromatin fiber traces in each cellular state, pairwise inter-probe distances were calculated, a threshold applied for distances below 250 nm and plotted as a “contact frequency” map (Figure 1E; see also Figure S1B for average pairwise distances). Both in hESCs and CNCCs, the *SOX9* TAD and flanking TADs were clearly observed, matching those previously identified by Hi-C^40^ (Figures 1E, upper and S1D). However, pronounced cell-type-specific changes in topology were seen within the *SOX9* TAD. In hESCs, the *SOX9* promoter preferentially interacted with the telomeric side of the TAD, with few long-range interactions across the domain and the presence of smaller blocks of interacting chromatin, reminiscent of the sub-TAD structures (Figure 1E, upper).^40^^,^^41^ By contrast, ORCA contact frequency maps for CNCCs revealed nearly uniform interactions across the *SOX9* TAD, with the exception of two striking stripe-like features emanating from the ORCA probe set overlapping the *SOX9* promoter and the E1.35 region (Figure 1E, lower—see arrows). These stripes and increased interactions of *SOX9* with the centromeric portion of the domain were further highlighted in the subtraction maps between hESCs and CNCCs (Figures 1F, upper and S1C). Taking advantage of the single-trace nature of the ORCA data, we performed bootstrapping analysis to determine which changes were statistically significant. This analysis further highlighted the switch from short-range interactions on the telomeric side of the *SOX9* TAD in hESCs to long-range TAD-spanning interactions in CNCCs (Figure 1F, lower, high-confidence maps showing pixels with confidence interval > 95% by bootstrapping).

The single-trace nature of the ORCA data allows us to quantify not only pairwise but also multiway interactions of the *SOX9* promoter region with the EC1.45, E1.35, and EC1.25 regions at the PRS locus. The median distance between the PRS ECs and the *SOX9* promoter was significantly decreased in CNCCs for all pairwise comparisons (Figure 1G, p < 0.001, K-S test). Furthermore, the frequency of promoter proximity < 250 nm to the EC1.45, E1.35, and EC1.25 elements was increased around 2-fold in CNCCs to 31.2%, 35.9%, and 27.3%, respectively (Figure 1G, insets). When considering all promoter-EC interactions, the proportion of ORCA traces for which the promoter is proximal to any of the three PRS locus elements increases to 60.9% in CNCCs, from 33.0% in hESCs. The majority of the increases are accounted for by an increase in multiway interactions involving more than one of the PRS locus elements (Figure 1H). Together, this suggests that in CNCCs, the *SOX9* promoter region frequently comes into close proximity with the ECs, perhaps involving simultaneous contacts with multiple distal elements (Figure S1E, compare right to left).

To determine whether the observed decreases in pairwise distances across the *SOX9* TAD translate to a smaller volume for the domain, we calculated the radius of gyration for hESCs and CNCCs across all observed traces. This analysis revealed that at the population level, there is a small but significant decrease in the radius of gyration (p < 0.001, Wilcoxon rank sum test) for the *SOX9* domain in CNCCs compared with hESCs (Figure 1I). However, this decrease was relatively modest (the overall radius of gyration changed only by around 13 nm) compared with the changes in pairwise distances within the domain, which were often greater than 100 nm (Figures 1G and S1C). Together, ORCA imaging revealed pronounced changes in the *SOX9* locus topology during neural crest differentiation, with large changes in pairwise interactions within the TAD, whereas a small change was observed in the overall volume of the domain.

### Locus topologies sampled by individual chromatin fibers are not unique to a given cell type, but certain conformations are more common in hESCs or CNCCs

**Figure 2:**
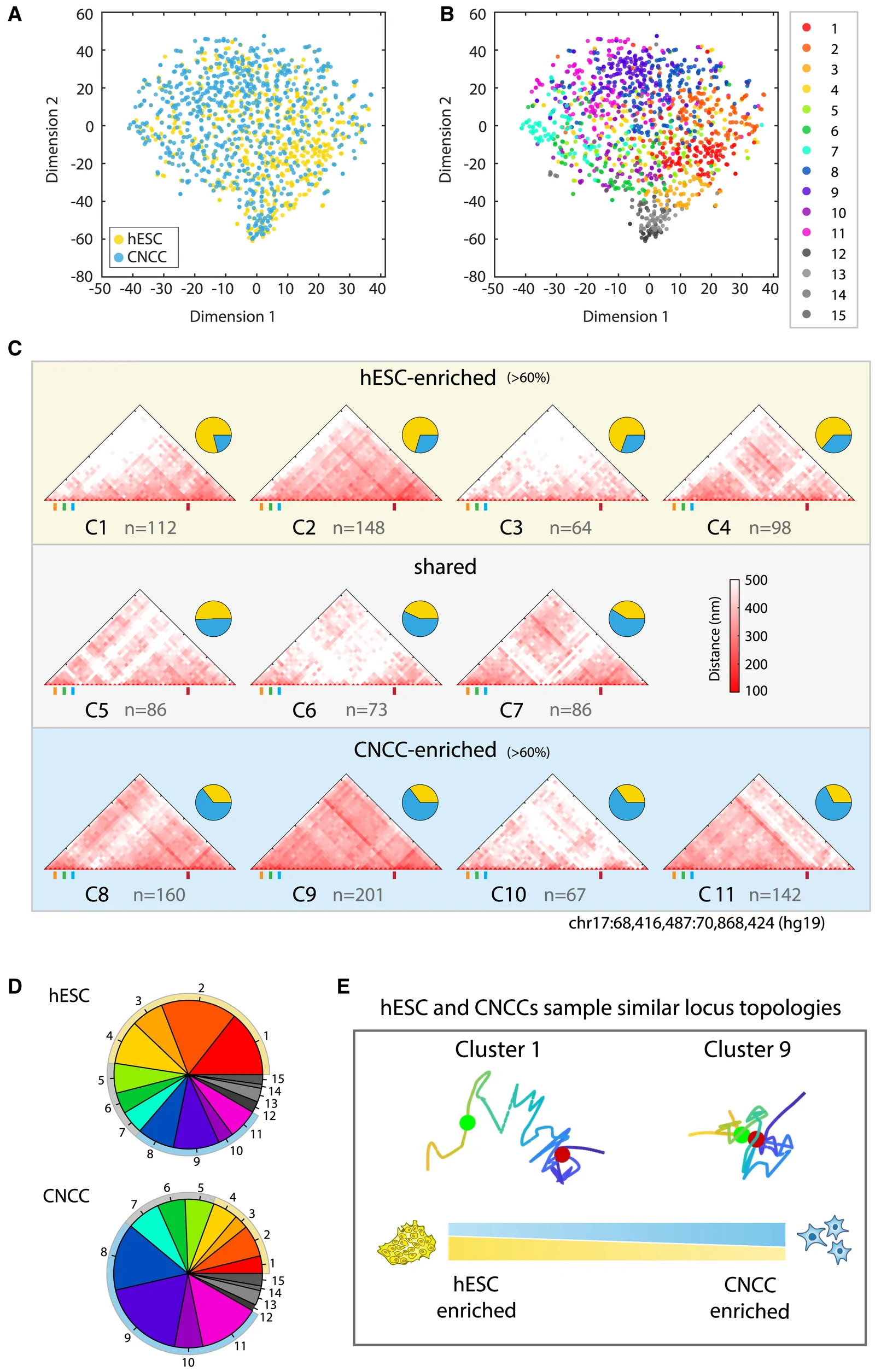
Distinct 3D domain conformations are enriched for, yet shared between, hESCs and CNCCs (A) tSNE plot representing for *SOX9* promoter pairwise distances with the *SOX9* TAD (Figure 1). (B) As (A), with 15 K-means clusters. Cluster colors are depicted on the right. (C) Absolute pairwise distance maps combining traces for each cluster from (B). Pie charts: proportions of traces for each cluster from hESC (yellow) or CNCC (blue). (D) Pie charts showing proportions of traces in each K-means cluster for hESC (upper) and CNCC (lower). (E) Schematic illustrating shared locus topology between cell types. MDS traces highlight exemplar hESC or CNCC-enriched structures. See also Figure S2.

Our initial analysis of the combined population heatmaps of *SOX9* locus topology revealed a distinct locus architecture for hESCs compared with CNCCs (Figure 1E). We therefore sought to leverage the single-trace nature of our dataset to distinguish between two plausible hypotheses of how these distinct architectures can arise at the population level: (1) through the presence of chromatin conformations unique to each cell type or (2) through cell-type-specific changes in the frequency of the conformations commonly sampled by both cell types. We restricted our analysis to the *SOX9* TAD and first focused on all pairwise distances for the 42 barcodes across this region. Using a t-distributed stochastic neighbor embedding (tSNE) analysis, we observed no clear separation or clustering for the two cell states (Figure S2A). We reasoned that many regions within the TAD may not contribute to cell-type specificity of the locus structure. We therefore reduced our analysis to only *SOX9* promoter pairwise distances to the surrounding TAD and observed a slight separation between hESC and CNCC traces on the tSNE plot. However, the overall topological conformations sampled by the promoter in both cell types appear to be highly overlapping (Figure 2A).

To explore whether certain locus conformations are more frequent in one cell type or the other, we performed K-means clustering of the *SOX9* locus topology. Three clusters were determined from the hESC-CNCC pairwise distance data (cluster number determined by elbow analysis) and visualized on the tSNE plot (Figures S2B and S2C). One of the three clusters, cluster 2, was relatively hESC-enriched (61% of traces), whereas cluster 1 was CNCC-enriched (56% of traces). Plotting the ensemble average heatmaps for these clusters revealed that cluster 1 showed uniform interactions across the TAD with two clear stripes, reminiscent of the ensemble map from CNCCs, whereas cluster 2 showed sub-TAD-like structures and enrichment of promoter-proximal interactions, reminiscent of the ensemble map from hESCs (Figure S2D).

To further sub-stratify distinct classes of structures within the *SOX9* TAD, we allowed for a larger number of clusters (15) and analyzed 11 clusters that had greater than 50 traces within them (Figures 2B and S2E, clusters 1–11). The 11 clusters occupied distinct regions of the tSNE map, with 4 clusters enriched in hESC and 4 enriched in CNCC (Figures 2C, 2D, and S2E). The remaining 3 clusters appeared to be present at comparable frequencies in both cell states. hESC-enriched ensemble heatmaps exhibited features reminiscent of the hESC population structure, including distinct sub-domain boundaries (e.g., clusters 1 and 2). By contrast, the CNCC-enriched clusters exhibited domain-wide interactions with the presence of the *SOX9* promoter stripe, and in some cases, also the E1.35 stripe (e.g., clusters 8 and 9) (Figure 2C). Taken together, our results are consistent with a model, whereby the *SOX9* promoter samples a similar constellation of locus topologies in hESCs and CNCCs, but certain conformations are more common in one of the cell types, resulting in substantial cell-type-specific differences in ensemble average contact maps (Figure 2E).

### The PRS locus E1.35 element regulates *SOX9* expression in the absence of enhancer activity

**Figure 3:**
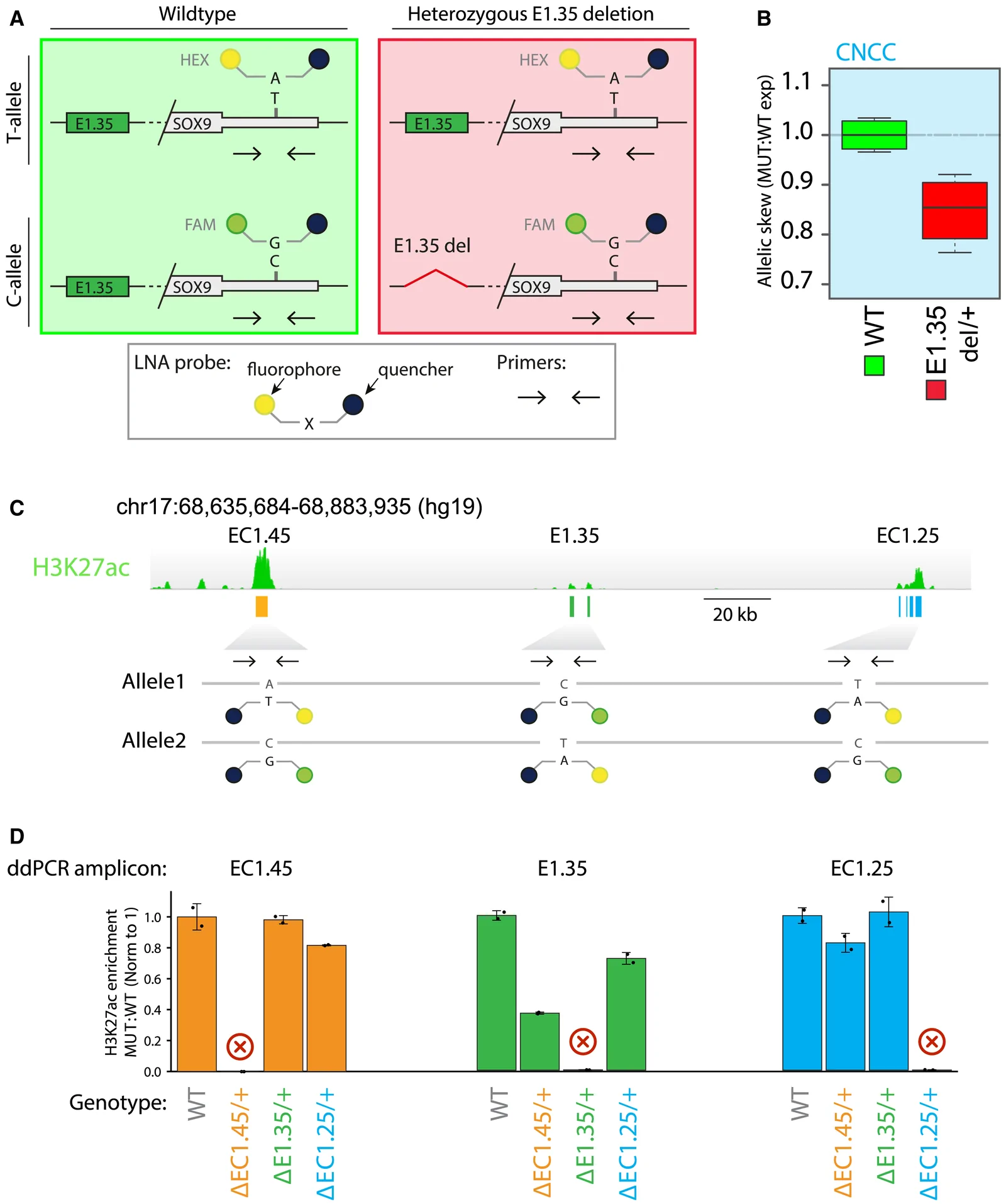
Ablation of E1.35 element affects *SOX9* expression but does not impact the activity of flanking enhancer clusters, as measured by active histone modifications (A) Schematic of allele-specific RT-ddPCR highlighting allele-specific SNPs and LNA probes for wild-type and heterozygous E1.35 deletion cell lines. (B) RT-ddPCR for wild-type and E1.35 heterozygous deletion, plotting mutant/wild-type expression, wild-type normalized to 1. (C) Schematic of allele-specific ddPCR for H3K27ac ChIP-ddPCR at EC1.25, E1.35, and EC1.45. (D) ChIP-ddPCR for H3K27ac at EC1.45, E1.35, and EC1.25 from wild-type and heterozygous deletions of EC1.45, E1.35, or EC1.25 CNCCs. Ratios of mutant to wild-type ratio signals are plotted, with wild-type normalized to 1. Error bars represent SD. See also Figure S3.

The most distinctive features of the CNCC contact frequency ensemble maps are two intersecting stripes at the ORCA barcodes that overlap with either the *SOX9* promoter region or the E1.35 regulatory element far upstream of *SOX9* (Figures 1E and 2C). In our previous work, E1.35 did not appear to have an autonomous regulatory capacity in luciferase assays and mouse LacZ transgenic reporter assays,^36^ suggesting that it may not function as an enhancer (Figures S3A and S3B). The apparent presence of a stripe feature at E1.35 led us to revisit a role for this region in the modulation of *SOX9* expression. To detect changes in *SOX9* expression on E1.35 ablation, we leveraged allele-specific digital droplet PCR (ddPCR), which enables sensitive detection of even small changes in expression by the direct comparison of gene expression from one allele (e.g., E1.35 mutant) versus the other (e.g., wild type) in the exact same cells. We performed ddPCR in this manner for two wild-type and two independent E1.35 heterozygous deletion lines during CNCC differentiation and observed a ∼20% reduction of gene expression on E1.35 loss (Figures 3A and 3B), an effect comparable with the expression reduction we have previously observed for EC1.25 deletion using the same ddPCR assay^36^ (Figure S3C). Therefore, despite its lack of canonical enhancer reporter activity, the E1.35 element contributes to *SOX9* regulation in CNCCs.

Given the genomic location of E1.35 between two strong ECs, we first hypothesized that E1.35 may act to facilitate or boost EC1.45 and/or EC1.25 activity, similar to facilitator elements recently described at the alpha globin locus.^44^ If E1.35 were acting to boost the activity of the flanking ECs, loss of E1.35 would reduce the activities of EC1.45 and/or EC1.25. We reasoned that H3K27ac levels could act as a proxy for enhancer activity and thus designed allele-specific probes and ChIP-ddPCR primers for each of the three regulatory elements. We performed H3K27ac ChIP-ddPCR in CNCCs that were either wild type or had a heterozygous deletion for each regulatory region (Figure 3C). Ablation of EC1.45 caused a small reduction in the H3K27ac ChIP signal for the EC1.25 region and a large (∼65%) reduction in the signal for the E1.35 element. Similarly, deletion of EC1.25 caused a small reduction in the signal at EC1.45 and a slightly larger impact on the E1.35 H3K27ac signal (Figure 3D). Thus, despite the ∼100 kb distance from E1.35 to each flanking EC, deletion of either EC1.45 or EC1.25 results in diminished H3K27ac at E1.35. By contrast, deletion of E1.35 had no impact on the H3K27ac signal for either EC1.45 or EC1.25. This result indicates that E1.35 does not act as a booster of PRS locus enhancer activity, at least as measured by their H3K27ac enrichment levels.

### SSEs at E1.35 and *SOX9* promoter mediate stripe formation and facilitate domain-wide interactions

**Figure 4:**
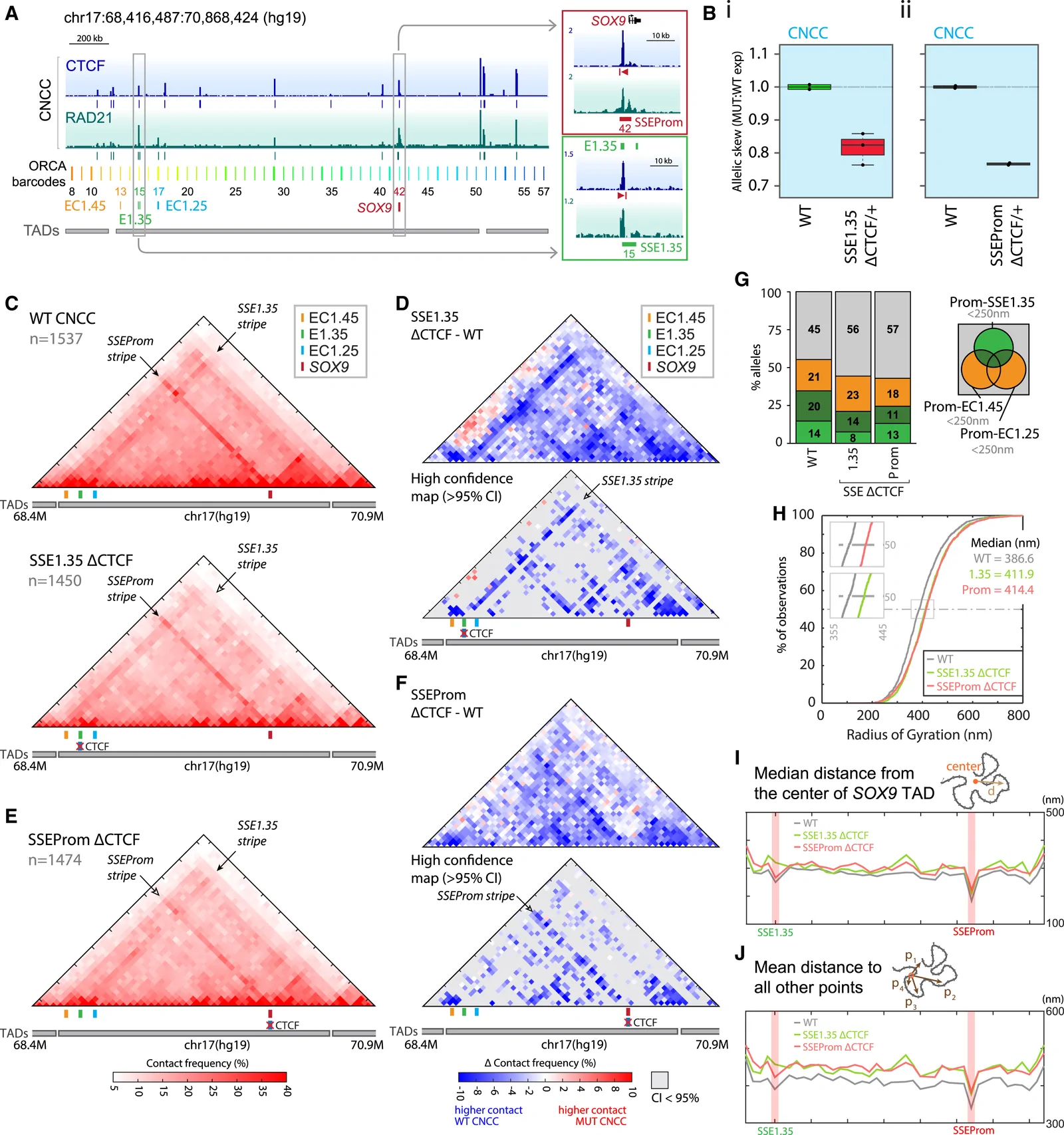
SSEs are required for TAD-spanning stripes, robust *SOX9* expression, and central positioning within TAD (A) CTCF and RAD21 ChIP-seq for CNCCs across *SOX9* TAD. ORCA barcodes 8–57 indicated (chr17:68416487:70868424, hg19). (B) RT-ddPCR for (i) E1.35 (SSE1.35) CTCF heterozygous deletion and (ii) SSEProm CTCF heterozygous deletion, compared with wild-type, plotting mutant/wild-type expression, wild-type normalized to 1. (C) ORCA contact frequency maps for pairwise distances < 250 nm for wild-type (upper) and E1.35 CTCF deletion (SSE1.35 ΔCTCF) CNCCs (lower). (D) Upper, contact frequency subtraction maps for E1.35 CTCF deletion (SSE1.35 ΔCTCF) compared with wild type. Lower, high-confidence map, confidence interval > 95%. (E) As (C), for SSEProm CTCF deletion CNCCs (SSEProm ΔCTCF). (F) As (D), for SSEProm ΔCTCF CNCCs. (G) Percentage of traces with promoter proximity to EC1.45, SSE1.35, and/or EC1.25 including multiway interactions for wild-type, SSE1.35 ΔCTCF and SSEProm ΔCTCF CNCCs. (H) Cumulative distribution function plots of the radius of gyration for the *SOX9* TAD in wild-type, SSE1.35 ΔCTCF and SSEProm ΔCTCF CNCCs. (I) Median distances from the center of the TAD to each point across the domain, averaged across all traces and plotted for each position across the locus. (J) Mean distance from a given point to all other points across the domain, plotted for each position across the locus. See also Figures S4 and S5.

We next hypothesized that E1.35 may play a structural role and influence *SOX9* expression by impacting locus topology, for example by acting as a tethering element between enhancers at the PRS locus and the *SOX9* promoter.^45^ Indeed, the TAD-spanning stripe originating from E1.35 in CNCCs may support this theory (Figure 1E). Stripe-like patterns (also known as lines or flares) have been observed at many genomic locations by Hi-C-based studies^5^^,^^39^^,^^46^^,^^47^ and have been proposed to form through the process of active loop extrusion stalled by barriers such as CTCF bound in a convergent orientation.^20^^,^^22^^,^^23^ Utilizing our published CTCF ChIP-seq data, we observed strong CTCF binding at the E1.35 element and proximal to the *SOX9* promoter, with the two CTCF sites arranged in a convergent orientation (Figure 4A). Notably, the E1.35-associated and promoter-proximal CTCF sites are not the sole TAD-internal sites bound by CTCF at the locus. However, these two CTCF sites appear to be associated with the highest level of RAD21 binding among the TAD-internal sites, consistent with their potential role as strong barriers to loop extrusion (Figure 4A).

To test whether CTCF binding is necessary for the contribution of E1.35 to *SOX9* expression, we generated and validated heterozygous hESC deletion lines in which the CTCF site within E1.35 was ablated (E1.35 ΔCTCF/+, Figures S4A and S4B). Subsequent analysis of *SOX9* allelic expression in CNCCs revealed that the loss of the CTCF binding site within E1.35 phenocopies the expression defect seen for the deletion of the entire element, with around 20% reduction (Figure 4Bi). To interpret the impact of E1.35 CTCF site ablation on *SOX9* gene expression, we next generated two different homozygous CTCF site mutant lines for subsequent ORCA imaging (E1.35 ΔCTCF, Figure S4C). We first performed CTCF and RAD21 ChIP-seq analyses from the E1.35 ΔCTCF cells and their wild-type counterparts and confirmed that: (1) CTCF and RAD21 bindings are lost from E1.35 and (2) no ectopic CTCF or RAD21 peaks appear at the locus on E1.35 CTCF site deletion (Figure S4D). ORCA imaging revealed a complete loss of the PRS locus-associated architectural stripe in the E1.35 ΔCTCF cells, and an overall reduction in contact frequency across the domain (Figures 4C and S5A). Subtraction of the E1.35 ΔCTCF contact frequency map compared with wild-type CNCCs emphasized these differences, with a large reduction in contact frequency observed along the E1.35 stripe and a general reduction in contact frequency across the TAD (Figure 4D; see arrow in the lower panel and Figure S5B). We therefore conclude that E1.35 functions as a structural element that contributes to gene expression, stripe formation, and long-range contacts across the locus in a CTCF site-dependent manner. Going forward, we will therefore refer to this element as “stripe-associated structural element 1.35” or SSE1.35 to emphasize the role of this region plays in regulating both domain folding and facilitating target gene expression. Of note, loss of the SSE1.35 stripe does not significantly impact the promoter-associated stripe, the location of the *SOX9* TAD boundaries, or inter-TAD contacts from the *SOX9* TAD, as highlighted by the high-confidence subtraction map (Figure 4D).

To explore the role of the second, promoter-associated stripe at the *SOX9* locus, we again ablated the associated CTCF binding site around 2.2 kb upstream of the *SOX9* TSS in a heterozygous or homozygous setting (SSEProm ΔCTCF/+ and SSEProm ΔCTCF cell lines, respectively; Figures S4A–S4D). Quantification of allele-specific *SOX9* expression in SSEProm ΔCTCF/+ CNCCs revealed a greater than 20% expression reduction from the CTCF-site ablated allele compared with the wild-type allele (Figure 4Bii). ORCA imaging of the SSEProm ΔCTCF cells revealed a reduction in TAD-wide contact frequency, with the stripe emanating from the promoter weakened, although in this case not fully lost (Figures 4E and S5C). Subtraction contact frequency maps and bootstrapping confirmed these changes (Figures 4F and S5D). Notably, CTCF and RAD21 ChIP-seq analyses revealed loss of CTCF, but only partial loss of RAD21 from the promoter in SSEProm ΔCTCF cells, suggesting a CTCF-independent contribution to a loop extrusion barrier at the promoter (Figure S4D). Together, we demonstrate that deletion of a CTCF binding site at the TAD-internal structural elements SSE1.35 or SSEProm ablates or weakens, respectively, the associated architectural stripe leading to a reduction of contacts across the domain (Figures 4C–4F).

To explore the mechanisms causing a reduction of *SOX9* transcription on ablation of either of two structural CTCF sites, we focused on enhancer-promoter proximity for the PRS locus ECs and the *SOX9* promoter region. Deletion of either CTCF site caused a significant increase in SSE1.35-promoter distances, measured by ORCA imaging (Figure S5E, p < 0.001, K-S test). By contrast, only promoter-associated CTCF site deletion (at SSEProm), but not SSE1.35 CTCF deletion, caused a significant shift in the EC1.45-promoter and EC1.25-promoter pairwise distances (Figure S5E, p < 0.01, K-S test). However, a general trend toward a reduction in pairwise contact frequency was observed for both CTCF deletions (Figure S5E, insets). Furthermore, the multiway interactions of the *SOX9* promoter to the three PRS locus regulatory elements were reduced in both SSE1.35 ΔCTCF and SSEProm ΔCTCF lines (Figure 4G, additional biological replicates shown in Figure S5F). Together, CTCF-bound SSEs appear to contribute to gene expression by facilitating domain-wide contacts and consequently promote multiway interactions between the PRS locus elements and the *SOX9* promoter.

### Central positioning of SSEs within the 3D locus topology

One possible explanation for how SSE1.35 and SSEProm facilitate domain-wide contacts is that they are situated centrally within the 3D domain structure (although, importantly, these elements are not in the middle of the TAD on the genomic map). Indeed, loss of CTCF binding is associated with a significant increase in the median radius of gyration (p < 0.001, Wilcoxon rank sum test) for both SSE1.35 ΔCTCF (6.5%) and SSEProm ΔCTCF (7.2%) (Figure 4H), indicating a decompaction of the *SOX9* locus in both ΔCTCF lines. The average structures derived from our ORCA data in CNCCs (Figure S1E) seem to support a central location of the two structural elements. The absolute distance measurements that can be quantified using single-chromatin fiber ORCA imaging allow us to test this hypothesis. To investigate the 3D location of SSE1.35 and SSEProm within the TAD, we plotted the median distance to the geometrical center of the TAD for all imaged regions across the domain (Figure 4I). We observed that SSE1.35 and SSEProm are the closest points to the geometrical TAD center and that deletion of the CTCF binding site at SSE1.35 causes SSE1.35 to move away from the geometrical TAD center (Figure 4I). The central positioning of the two SSEs suggests that both SSEs are close to all other regions within the *SOX9* TAD. Indeed, plotting the mean pairwise distance for each imaged region to all other points in the *SOX9* TAD revealed that the shortest mean pairwise distances were for SSE1.35 and SSEProm (Figure 4J). Moreover, on SSE1.35 CTCF deletion, all mean pairwise distances across the domain are increased, further supporting the idea that this structural element promotes TAD compaction (Figure 4J). Interestingly, although deletion of the SSEProm CTCF site leads to a similar TAD decompaction and increase in mean pairwise interactions across the TAD (Figures 4H and 4J), the *SOX9* promoter remains both the closest point to the geometrical TAD center and to all other regions within the TAD even in the absence of the SSEProm CTCF site (Figures 4I and J). This suggests that in contrast to SSE1.35, CTCF binding is not the only feature that promotes the central positioning of the *SOX9* promoter. Together, these data reveal that SSE1.35 and the *SOX9* promoters (SSEProm) are situated centrally within the 3D domain structure, facilitate domain-wide interactions, and promote the formation of a compact structure across the TAD.

### A multi-loop model better explains the topological features of the *SOX9* locus than a reel-in model

**Figure 5:**
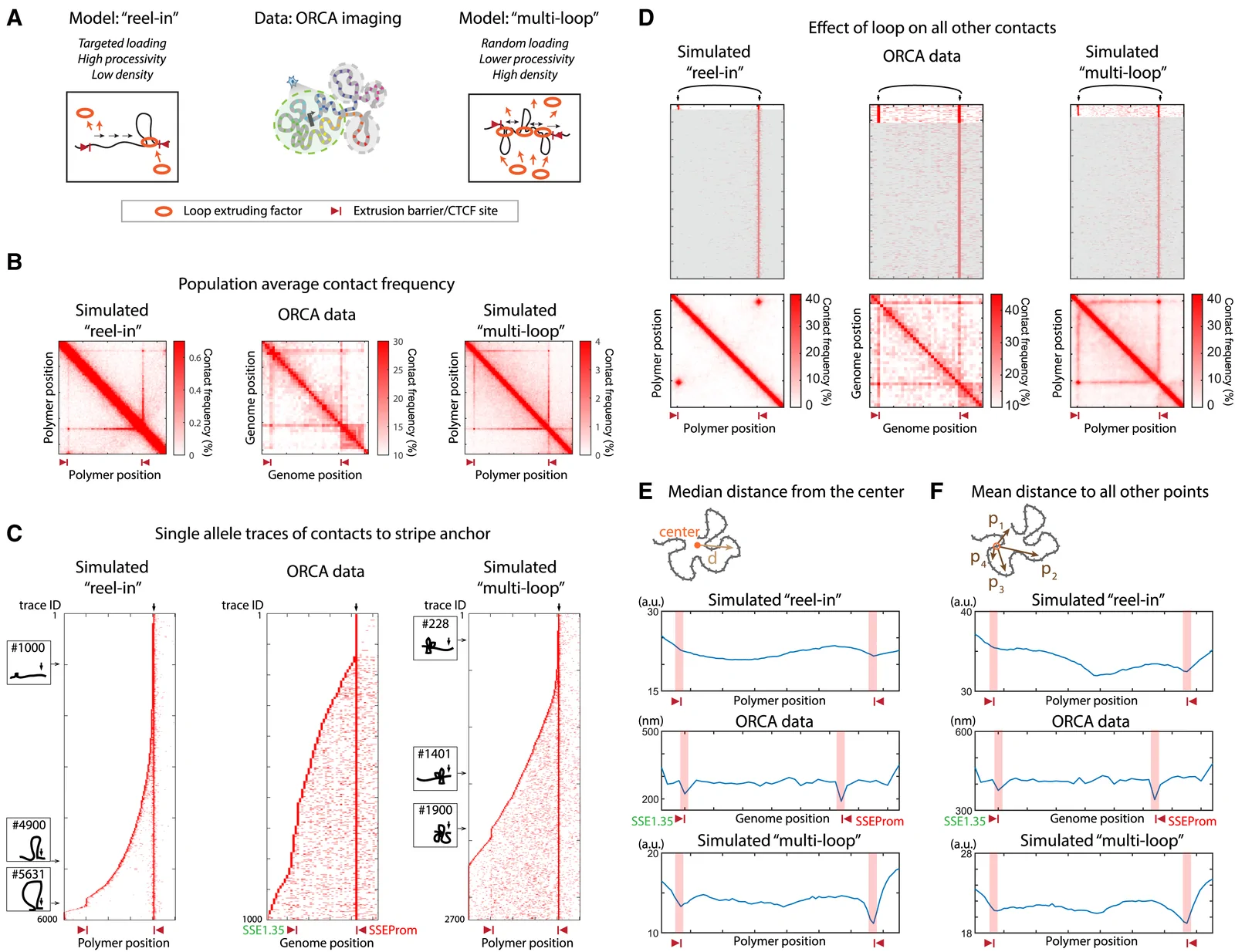
A multi-loop model best fits the observed stripes and central positioning of SSEs at the *SOX9* locus (A) Schematics of loop extrusion-based models to explain *SOX9* locus topology. Left, reel-in model: targeted cohesin loading at one site near a loop extrusion barrier with low density of loading and a long processivity. Right, multi-loop model: random cohesin loading between two loop extrusion barriers at high density with short processivity. (B) Population-level contact frequency maps from polymer simulations of the reel-in model (left) and multi-loop model (right), compared with ORCA data from CNCCs (middle, data from Figure 1). (C) Single allele traces, showing all contacts with the right stripe anchor, sorted by most distal contact. Cartoons of exemplar traces for reel-in and multi-loop models (see Figures S5A and S5B). Arrow: viewpoint at the right-hand side stripe anchor. (D) Heatmap of traces for pairwise contacts from one stripe anchor across the domain ordered by interactions with a specific point (black arrows) (upper). Population-level contact frequency maps from traces showing contacts in the upper traces (lower). (E) Median distances from the center of the TAD/domain to each point across the domain, averaged across all traces and plotted for each position across the locus. (F) The mean distance from a given point to all other points across the domain is plotted for each position across the locus. Red triangle: stripe anchor/loop extrusion barrier. See also Figure S6.

To gain mechanistic insight into the processes underlying the central positioning and architectural stripe formation of the two SSEs, we turned to polymer simulations. Polymer models including the loop extrusion and phase-separation frameworks have been proposed as physical mechanisms driving the spatial organization of chromatin.^48^^,^^49^^,^^50^ In particular, based on experimental data and polymer simulations, it has been proposed that architectural stripes are an ensemble or population feature of the dynamic process of loop extrusion by cohesin via a so-called reel-in model.^39^ In this model, cohesin is loaded at active enhancers or promoters near one or more CTCF sites.^5^^,^^39^^,^^47^^,^^51^ Soon after loading, CTCF arrests a single extrusion subunit, whereas its partner continues to reel-in DNA in the opposite direction, forming a continuously growing single loop until a second extrusion barrier is encountered (Figure 5A).

Our ORCA imaging dataset at the *SOX9* locus provides a unique opportunity to build and compare polymer models with experimental data at both ensemble and single-chromatin fiber levels. To this end, we built and tested two models using the “open2c/polychrome” framework.^52^ In both models, we introduced two strong domain-internal barriers to loop extrusion, corresponding to SSEs positions within the domain, but the two models substantially differ in the parameters governing the behavior of the loop-extruding factor (LEF/cohesin). In the first model, we introduced parameters consistent with the previously described reel-in model,^39^ namely LEF/cohesin was loaded at a low density, with loading occurring at a single site adjacent to a strong loop barrier, and LEF/cohesin was highly processive, allowing it to remain bound to DNA and extrude for an extended period. In this configuration, the polymer forms a single loop for each iteration of LEF/cohesin binding (Figures 5A, left and S6A). In a second model, which we call the multi-loop model, LEF/cohesin was loaded randomly at any location between the two loop barriers, and loading density was high; however, LEF/cohesin processivity was relatively low (Figures 5A, right and S6B). When two extrusion barriers are included across a simulated region, both models result in the formation of two stripes in the ensemble-level maps. These population-level heatmaps are reminiscent of the architectural stripes we observed at the *SOX9* locus by ORCA imaging (Figure 5B).

We next explored predictions of these two models at a single-chromatin fiber level as the reel-in and multi-loop models imply different molecular dynamics during the loop extrusion process. To compare the two models with our experimental measurements, we looked at interactions from one stripe anchor to all points across the domain, sorting the ORCA traces based on the most distal interaction from the stripe anchor. We observed multiple intervening contact points from single traces even at a strict threshold for “contact” (<150 nm) (Figures 5C, middle and S6C). The ensemble maps from combined single traces that show contact between the stripe anchor and an arbitrary point revealed a domain-spanning stripe (Figure 5D, middle). These experimental measurements were much better recapitulated by the simulated multi-loop model, where multiple contact points and a population-level stripe were observed (Figures 5C, right and 5D, right). By contrast, in the simulated reel-in model, the stripe anchor only contacts one point in the domain in most cases (as seen by the white space between the right anchor and the extruding loop in the individual traces, Figure 5C, left, and by a “dot”-like contact in the ensemble map, Figures 5D, left and S6D).

Finally, we investigated whether the central position of the two structural elements within the 3D domain structure can be recapitulated by either of the two polymer models. Similar to our observations from the experimental data, in the multi-loop model simulations, the loop barriers show the shortest distance to the center of the domain and to the other points within the domain (Figures 5E and 5F, compare middle and lower panels). Indeed, in the multi-loop model simulations, multiple LEFs/cohesins load onto the polymer, stall at loop barriers, and consequently result in the loop barriers being frequently positioned in the center of the domain (as seen for a representative simulation, Figure S6E). This is a unique feature of the multi-loop model and not observed in the reel-in model simulations (Figures 5E and 5F, upper). In summary, our experimental results are better explained by the multi-loop model, suggesting that at large regulatory domains, such as the *SOX9* locus, ultra-long-range interactions are facilitated both by the presence of TAD-internal structural elements and the distributed loading of multiple cohesin molecules. As such, the *SOX9* promoter and its distal enhancers are rarely contained within the single cohesin-mediated loop but rather are joined by a bridge of multiple cohesin molecules stalled against one another.

## Discussion

**Figure 6:**
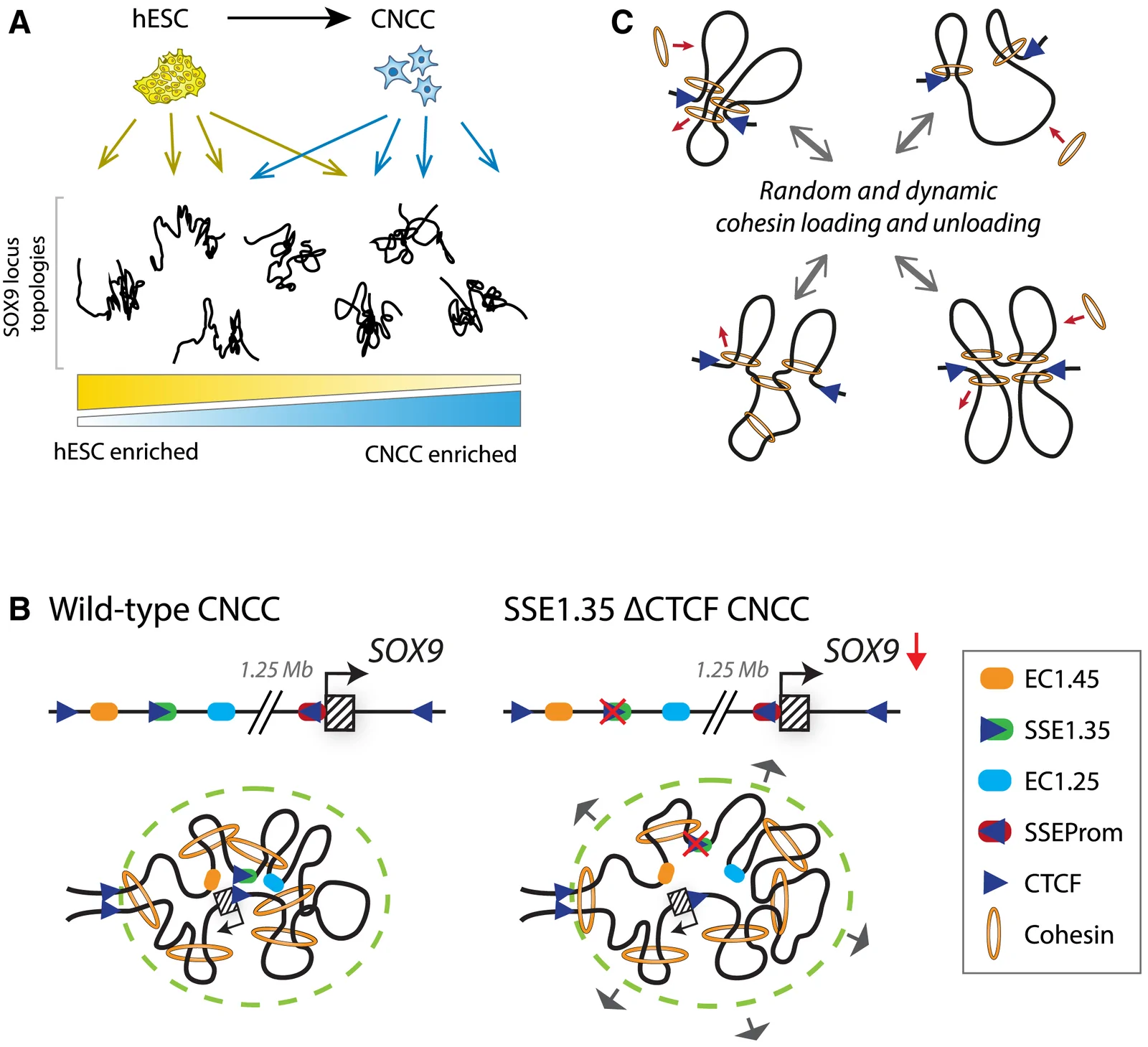
Schematic models of changes in *SOX9* locus folding during differentiation, and the role of SSEs and multi-loop topologies (A) Ensemble-average differences in *SOX9* locus topology in hESC and CNCC arise through cell-type specific changes in frequency of conformations commonly sampled by both cell types. (B) SSEs SSE1.35 and SSEProm are bound by CTCF and located in the geometrical center of the 3D domain (left panel). Mutation of the SSE results in domain decompaction and decreased domain-wide contacts, leading to diminished *SOX9* expression (right panel). (C) ORCA data and polymer simulations support a multi-loop model of *SOX9* locus topology. In this model, the multi-loop formation is mediated by the loop extrusion process, with two SSEs serving as strong loop extrusion barriers. Important parameters of the model include random loading of cohesin and allowing multiple cohesin complexes to extrude chromatin within the domain simultaneously, with frequent collisions.

Through tracing the 3D locus topology of the *SOX9* regulatory domain in hundreds of cells, we revealed significant changes in the internal TAD organization during differentiation of hESCs to craniofacial progenitors, CNCCs. This observation agrees with Hi-C-based studies that have demonstrated that although the overall TAD structure is typically well-preserved across cell types,^3^^,^^4^ cell-type-specific conformational changes can be observed within TADs.^46^^,^^47^^,^^53^^,^^54^^,^^55^ The single-chromatin fiber nature of our ORCA datasets enabled us to probe variations in the locus architecture within and between cell types. Despite divergent population-average structures, individual hESCs or CNCCs could not be distinguished based on the structure alone. Instead, ensemble-average differences in locus topology arise through cell-type-specific changes in the frequency of commonly sampled 3D conformations (see Figure 6A for model schematic), highlighting the flexible nature of the locus topology across a population of cells.

One of the most striking features of the *SOX9* locus ensemble interaction frequency maps in CNCCs was the two TAD-spanning stripes. We identified two SSEs, which promote stripe formation, are bound by CTCF, and facilitate expression from the *SOX9* gene. We note parallels between the two SSEs identified here and tethering elements recently reported in *Drosophila* development.^45^ Both SSEs and Drosophila tethering elements are associated with open chromatin, do not overlap with TAD boundaries, and impact gene expression when ablated, despite not having autonomous enhancer activity. However, there are also many notable distinctions, including that the *SOX9* locus SSEs are CTCF dependent and associated with stripes rather than points/dots on contact frequency maps. This suggests that the Drosophila tethering elements may form relatively frequent loops between two specific elements, whereas the *SOX9* locus SSEs described here facilitate broad domain-wide interactions.

Promoter-proximal CTCF binding has also been demonstrated in mouse embryonic stem cells and neural progenitor cells and in human cancer cell lines and is shown to facilitate contacts between promoters and enhancers that lie between the CTCF sites.^29^^,^^30^ By contrast, at the *SOX9* locus, two CTCF-bound SSEs sit in between (rather than flanking) the strongest distal enhancer (EC1.45) and its target gene. Therefore, the *SOX9* locus SSEs appear to mediate enhancer activity from both upstream and downstream of their long-range interaction. Notably, this is also distinct from the regulatory neighborhoods that appear to act through an insulation-based mechanism that brings enhancers and promoters into a shared domain.^28^ We propose that instead of promoting insulation or directionality, the *SOX9* locus SSEs facilitate the function of distal ECs in *SOX9* gene activation through enabling the formation of a multi-loop structure and compaction of the entire domain (see Figure 6B for model schematic). In turn, domain compaction driven by the SSEs facilitates more uniform contacts across the domain, allowing even very distal enhancers within the domain to act on the promoter. In agreement with our model, chromatin decompaction has also been observed following acute degradation of CTCF.^26^^,^^56^

We observe a hub-like conformation at the *SOX9* locus that features a high frequency of ORCA traces with the *SOX9* gene located close to the 3D center of the domain. By virtue of its central positioning, the promoter appears to be in an optimal location to sample interactions with all other regions across the domain, including all enhancers. The CTCF site at SSE1.35 also draws the distal region of the TAD into the center of the domain. The two SSEs are not entirely equivalent, however, as the central positioning of SSE1.35 appears less pronounced than for SSEProm, and both the central positioning and stripe are lost for SSE1.35 on CTCF site deletion. By contrast, the promoter-adjacent stripe is less dependent on CTCF binding, suggesting partial redundancy with other mechanisms mediating its formation. For example, RNA Pol II has also been shown to block cohesin movement on chromatin, thus serving as an additional extrusion barrier.^57^^,^^58^ Indeed, in contrast to the SSE1.35, deletion of the CTCF site at the promoter only partially diminishes cohesin occupancy, consistent with additional factors at the promoter contributing to the extrusion barrier function.

To explore how architectural stripes and regulatory hubs may form, we applied polymer simulations of the loop extrusion. Counter to an existing reel-in model that emphasized targeted loading of cohesin,^39^^,^^47^^,^^51^ we observed multiple interactions across the *SOX9* domain between SSE1.35 and the *SOX9* promoter. Our ORCA results and simulations therefore support a multi-loop model with two important parameters, first that cohesin complexes are loaded randomly between the two loop barriers and second that multiple cohesin complexes can extrude chromatin within the domain simultaneously but cannot proceed past one another. This implies that loop-extruding cohesin complexes are dense enough and processive enough at the *SOX9* locus to collide frequently. In further support of our model, recent work has demonstrated that polymer simulations using uniform cohesin loading more closely reproduced the experimental Hi-C maps than preferential loading of cohesin at the promoter.^57^ Furthermore, it has been estimated from Micro-C data that cohesin processivity is around 150 kb.^59^^,^^60^ This processivity would not be sufficient for a cohesin complex to extrude across the entire 1.35 Mb between the two barriers, as implied by the reel-in model. Reported measures of cohesin processivity are therefore more consistent with the multi-loop model. Moreover, the multi-loop model not only best fits our empirical data at the *SOX9* locus but also predicts the central positioning of the two SSEs within the regulatory domain. This suggests that the central positioning of SSEs is—at least in part—a consequence of the loop extrusion process.

We note certain parallels between the structural elements described here and the CTCF-anchored bridge sites described by Hafner et al.^56^ Both types of elements are composed of similar components, such as a CTCF site and stalling cohesin molecules, and both appear to enhance particularly long-range contacts through the formation of multi-loop structures. However, the SSE elements at the *SOX9* locus differ from the bridge elements in that they do not cross over any notable domain border and are associated with prominent stripes, whereas not all bridge sites form visible stripes on the ensemble maps. Our results also raise the question of why some, but not all, CTCF/cohesin peaks found inside TADs form stripes. One potential mechanism is that there are additional CTCF cofactors contributing to loop extrusion stalling and stripe formation. For example, MAZ has been proposed to function as a cofactor for CTCF insulation at boundaries.^61^ If some of these potential cofactors specifically inhibited cohesin unloading in a direction-dependent manner, they could preferentially lead to stripe formation. One other potential mechanism is that at the level of entire TADs, the density of cohesin might be different between distinct subnuclear environments. In our polymer simulation, multiple simultaneously extruding cohesins are required for stripe formation, and therefore, stripe formation may be limited to environments with a high local concentration of cohesin.

Overall, our ORCA data and polymer simulations suggest that the *SOX9* locus stripes are built from multiple loops that frequently tether the SSE1.35 element to the *SOX9* promoter via multiple cohesin bridges (see Figure 6C for the model schematic). For extreme long-range gene regulatory loci, such as at the *SOX9* locus, the multiple loop configuration provides a number of advantages, overcoming the low reported processivity of cohesin by multiple cohesin-mediated loops that compact the domain and enable faster promoter-enhancer interaction compared with single extruding factor scanning along the domain. The multiple cohesin configuration also buffers the effect of stochastic loading/unloading of cohesin, facilitating robust interactions. Together, the multi-loop model provides a conceptual framework for understanding how structural elements can enable domain-wide interactions, which in turn may facilitate robust regulation of gene expression, especially at large TADs with extremely distal enhancers.

Craniofacial development appears to be highly sensitized to alterations in levels of *SOX9* expression in CNCCs.^31^^,^^36^ Indeed, the 20% reduction in *SOX9* expression that is observed on the ablation of either SSE1.35 or SSEProm corresponds to a level of dysregulation that is sufficient to drive phenotypic changes in mouse models.^36^ Therefore, as more genome sequences become available from patients with PRS and related dysmorphologies in the future, we postulate it might be worthwhile to scan for mutations affecting CTCF binding at the two SSEs identified in this study.

### Limitations of the study

Although the proposed multi-loop model explains how the identified TAD-internal structural elements promote stripe formation and facilitate locus compaction and long-range gene regulation, our observations are limited to a single locus—the *SOX9*. It remains to be seen to what extent the multi-loop model is generalizable to other loci associated with architectural stripes and/or those at which principal enhancers regulate genes over ultra-long genomic ranges. We also acknowledge that we have not explored here alternative chromatin polymer models beyond the loop-extrusion framework, such as the String & Binders Switch (SBS) that models chromatin folding in the context of the phase separation.^49^^,^^62^ It would be of interest to test in the future the potential of such models to explain our experimental observations and to explore whether the combined loop extrusion and phase separation mechanisms can more robustly recapitulate ORCA data.

## STAR★Methods

### Key resources table

**Table:** 

REAGENT or RESOURCE	SOURCE	IDENTIFIER
**Antibodies**		

Rabbit polyclonal H3K27ac – ChIP	Active Motif	Cat# 39133; RRID:AB_2561016
Rabbit polyclonal CTCF – ChIP	Cell Signalling	Cat# 2899; RRID:AB_2086794
Rabbit polyclonal RAD21 – ChIP	Abcam	Cat# ab992; RRID:AB_2176601

**Chemicals, peptides, and recombinant proteins**		

mTeSR	Stem Cell Technologies	Cat# 85850
Matrigel Growth Factor Reduced (GFR) Basement Membrane Matrix	Corning	Cat# 356231
ReLeSR	Stem Cell Technologies	Cat# 05872
Collagenase IV	Gibco	Cat# 17104019
DMEM/F12 1:1 medium, with L-glutamine; without HEPES	GE Healthcare	Cat# SH30271.FS
Neurobasal Medium	Thermo Fisher Scientific	Cat# 21103049
Gem21 NeuroPlex Supplement With Vitamin A	Gemini Bio-Products	Cat# 400-160
N2 NeuroPlex Supplement	Gemini Bio-Products	Cat# 400-163
Antibiotic-Antimycotic (100X)	Gibco	Cat# 15240062
GlutaMAX Supplement (100X)	Life Technologies	Cat# 35050061
Recombinant Human FGF-basic (154 a.a.)	PeproTech	Cat# 100-18B
Animal-Free Recombinant Human EGF	Peprotech	Cat# AF-100-15
Bovine Insulin Powder	Gemini	Cat# 700-112P
Human Plasma Fibronectin Purified Protein	MilliporeSigma	Cat# FC01010MG
Accutase	Sigma-Aldrich	Cat# A6964-100ML
Bovine Serum Albumin (BSA), Fraction V—Serum Replacement Grade	Gemini Bio-Products	Cat# 700-104P
Recombinant Human/Murine/Rat BMP-2 (E.coli derived)	PeproTech	Cat# 120-02
CHIR-99021 (CT99021) HCl	Selleck Chemicals	Cat# S2924
DMEM/High glucose with L-glutamine, sodium pyruvate	Cytiva (formerly GE Healthcare)	Cat# SH30243.01
Sodium pyruvate	Life Technologies	Cat# 11360070
Ascorbic acid	Sigma-Aldrich	Cat# A4403-100MG
Dexamethasone	Thermo Fisher Scientific	Cat# AAA1759003
Recombinant Human TGF-β3	PeproTech	Cat# 100-36E
Y-27632 RHO/ROCK pathway inhibitor	Stem Cell Technologies	Cat# 72304
KnockOut DMEM	Gibco	Cat# 10829018
cOmplete, EDTA-free Protease Inhibitor Cocktail	MilliporeSigma	Cat# 11873580001
QuickExtract DNA Extraction Solution	Lucigen	QE09050
EvaGreen Dye	Biotium	Cat# 31000
RNasin Plus RNase Inhibitor	Promega	Cat# N2611
Maxima H Minus RT Transcriptase	ThermoFisher Scientific	Cat# EP0751

**Critical commercial assays**		

NEBNext Ultra II DNA Library Prep Kit for Illumina	New England BioLabs	Cat# E7645S
TRIzol Reagent	Invitrogen	Cat# 15596018
FuGENE 6	Promega	Cat# E2691
NEBNext Multiplex Oligos for Illumina kit	New England BioLabs	Cat# E7335S
AMPure XP	Beckman Coulter	Cat# A63881
Dynabeads Protein G for Immunoprecipitation	Invitrogen	Cat# 10004D
Qubit dsDNA HS Assay Kit	Invitrogen	Cat# Q32854
SuperScript IV VILO Master Mix with ezDNase Enzyme	Invitrogen	Cat# 11766050
Phusion Hot-start Master Mix	New England BioLabs	Cat# M0536S
DNA Clean & Concentrator-5	Zymo Research	Cat# D4013
HiScribe T7 Quick High Yield RNA Synthesis Kit	New England BioLabs	Cat# E2050S
DNA Clean & Concentrator-25	Zymo Research	Cat# D4005

**Deposited data**		

CTCF and RAD21 ChIP-seq	This paper	GEO: GSE223132
H9 hESC 10X Genomics linked-read sequencing	Long et al.^36^	Sequence Read Archive (SRA): PRJNA648128
ChIP-seq, ATAC-seq in ESCs and CNCCs	Long et al.^36^; Prescott et al.^63^	GEO: GSE70751 and GSE145327
Hi-C in H1-hESC	Akgol Oksuz et al.^40^	4DN data portal: 4DNESRJ8KV4Q
ORCA processed datasets	This paper	4DN data portal: 4DNESID7GI8N 4DNESNIOLISN 4DNESEQDL9UR 4DNESWPT973M 4DNESL3FF6MP

**Experimental models: Cell lines**		

Human: Female H9 human embryonic stem cells (hESCs)	WiCell	WA09; RRID: CVCL_9773
H9 *SOX9* ΔEC1.45/+	Long et al.^36^	N/A
H9 *SOX9* ΔE1.35/+	This paper	See STAR Methods and Methods S1
H9 *SOX9* ΔEC1.25/+	Long et al.^36^	
H9 *SOX9* SSE1.35 ΔCTCF/+	This paper	See Figures S4A–S4D and STAR Methods
H9 *SOX9* SSEProm ΔCTCF/+	This paper	See Figures S4A–S4D and STAR Methods
H9 *SOX9* SSE1.35 ΔCTCF	This paper	See Figures S4A–S4D and STAR Methods
H9 *SOX9* SSEProm ΔCTCF	This paper	See Figures S4A–S4D and STAR Methods

**Oligonucleotides**		

Primers for qRT-PCR and LNA probe	This paper	see Table S3

**Software and algorithms**		

Chromatin tracing analysis code	Alistair Boettiger	https://doi.org/10.5281/zenodo.7698979
Polymer simulation	Imakaev et al.^52^ & Alistair Boettiger	https://doi.org/10.5281/zenodo.7698987
Code for image analysis and executable scripts to simulate the “reel-in” and the “multi-loop” models	This study	https://doi.org/10.5281/zenodo.7698897
Benchling	Benchling [Biology Software]. (2017)	https://www.benchling.com/
The R package for Statistical Computing (v3.6.0)	R Core Team	https://www.r-project.org/
R Geomorph package	Adams and Otárola-Castillo^64^	https://cran.r-project.org/web/packages/geomorph/index.html
R hotelling.test function	James Curran and Taylor Hersh	https://cran.r-project.org/web/packages/Hotelling/Hotelling.pdf
Skewer	Jiang et al.^65^	https://github.com/relipmoc/skewer
bowtie2	Langmead and Salzberg^66^	http://bowtie-bio.sourceforge.net/bowtie2/index.shtml
Bedtools	Quinlan and Hall^67^	https://github.com/arq5x/bedtools2
bedgraphToBigWig	Kent et al.^68^	https://github.com/ENCODE-DCC/kentUtils
macs1.4	Zhang et al.^69^	https://github.com/taoliu/MACS
Cutadapt	Martin^70^	https://cutadapt.readthedocs.io/en/stable/
featureCounts (subread package)	Liao et al.^71^	http://subread.sourceforge.net/
Long Ranger (longranger-2.2.2)	10X Genomics	https://support.10xgenomics.com/genome-exome/software/pipelines/latest/what-is-long-ranger
Macs2 (macs2 2.1.1.20160309)	Zhang et al.^69^	https://github.com/taoliu/MACS
SeqPos (Cistrome Project)	Liu et al.^72^	http://cistrome.org/
Ggseqlogo	Wagih^73^	https://cran.r-project.org/web/packages/ggseqlogo/ggseqlogo.pdf
ggplot2	Wickham^74^	https://ggplot2.tidyverse.org/
UCSC	Kent et al.^75^	https://genome.ucsc.edu/
Samtools (v1.3.1)	Li et al.^76^	http://samtools.sourceforge.net/
Bioanalyzer 2100 Expert Software	Agilent	https://www.agilent.com/en/product/automated-electrophoresis/bioanalyzer-systems/bioanalyzer-software/2100-expert-software-228259
QuantaSoft Software	BioRad	https://www.bio-rad.com/en-us/sku/1864011-quantasoft-software-regulatory-edition?ID=1864011

**Other**		

Covaris sonicator E220	Covaris	N/A
QX200 Droplet Generator	BioRad	https://www.bio-rad.com/en-gu/sku/1864002-qx200-droplet-generator?ID=1864002
QX200 Droplet Reader	BioRad	https://www.bio-rad.com/en-us/sku/1864003-qx200-droplet-reader?ID=1864003
LightCycler 480	Roche	N/A
Bioanalyzet R	Agilent	https://www.agilent.com/en/product/automated-electrophoresis/bioanalyzer-systems/bioanalyzer-instrument/2100-bioanalyzer-instrument-228250

### Resource availability

#### Lead contact

Further information and requests for resources and reagents should be directed to and will be fulfilled by the lead contact, Joanna Wysocka (Wysocka@stanford.edu).

#### Materials availability

• Cell lines generated by the authors will be distributed upon request to other researchers.

### Experimental model and subject details

#### Culturing human embryonic stem cells (hESCs)

Human embryonic stem cells (hESCs) were purchased from ATCC (WA09, also known as H9; RRID: CVCL_9773). hESCs were fed every day with mTeSR (Stem Cell Technologies), grown on Matrigel Growth Factor Reduced (GFR) Basement Membrane Matrix (Corning) at 37°C and passaged every 5-6 days using ReLeSR (Stem Cell Technologies).

### Method details

#### Genome-editing of hESC cell lines using CRISPR/Cas9

Two edited hESC cell lines were generated previously, ΔEC1.45/+ and ΔEC1.25/+.^36^ Targeted deletion of E1.35 was performed in a similar manner: two targeting strategies were used with distinct homology arms and single guide RNA sequences (sgRNAs; see Methods S1 for plasmid sequences). Briefly, H9 hESCs were transfected using FuGENE 6 (Promega) with one of the two targeting constructs (containing Blasticidin selection cassette, flanked by FRT sites, and homology arms for either side of E1.35) along with a plasmid encoding Cas9 plus two sgRNAs that target sites either side of E1.35. Transfected hESCs were grown to confluency and split onto a new plate before selection with 1 mg/mL Blasticidin until all cells died on a mock/GFP transfected control well. Surviving colonies were picked into a 48-well plate, expanded, passaged, and screened for enhancer deletion using a genomic primer and a primer within the targeting cassette. Heterozygous enhancer deleted clones were transfected with a Flippase-expressing plasmid and clones screened for excision of the selection cassette by PCR. For screening, genomic DNA was extracted using QE buffer (Lucigen) and PCR was performed using Q5 polymerase (NEB). Heterozygous enhancer deletions were generated to facilitate allele-specific *SOX9* gene expression analysis. To generate SSE ΔCTCF cell lines, a plasmid encoding for Cas9 and a single sgRNA (targeting a CTCF site) was transfected into H9 hESCs. Following transfection, transfected cells were enriched by FACS and plated at low density on a 10cm dish. Colonies were picked into a 96-well plate, passaged once, and screened for CTCF site deletion by diagnostic digestion of a PCR product, then verified by Sanger sequencing. Both heterozygous and homozygous CTCF deletion lines were identified and expanded. To ensure clonality, selected clones were dilution-plated at single-cell density and colonies were picked and expanded. See Methods S1 and Figures S4B and S4C for the targeting plasmid and sgRNA sequences.

#### Differentiation of hESC to CNCCs

hESCs were differentiated to human cranial neural crest cells (CNCCs) as described previously.^36^^,^^63^ Briefly, hESCs were grown for 5-6 days until large colonies formed, which were disaggregated using collagenase IV and gentle pipetting. Clumps of ∼200 hESCs were washed in PBS and transferred to a 10cm Petri dish in neural crest differentiation media (NDM). NDM: 1:1 ratio of DMEM-F12 and Neurobasal, 0.5x Gem21 NeuroPlex Supplement With Vitamin A (Gemini, 400-160), 0.5x N2 NeuroPlex Supplement (Gemini, 400-163), 1x antibiotic/antimycotic, 0.5x Glutamax, 20ng/ml bFGF (PeproTech, 100-18B), 20ng/ml EGF (PeproTech, AF-100-15) and 5ug/ml bovine insulin (Gemini Bio-Products, 700-112P). After 7-8 days, cranial neural crest cells (CNCCs) emerged from neural spheres that had attached to the Petri dish. After 11 days, CNCCs were passaged onto fibronectin-coated 6-well plates using accutase and fed with neural crest maintenance media (NMM). NMM: 1:1 ratio of DMEM-F12 and neurobasal, 0.5x Gem21 NeuroPlex Supplement with Vitamin A (Gemini, 400-160), 0.5x N2 NeuroPlex Supplement (Gemini, 400-163), 1x antibiotic/antimycotic, 0.5x Glutamax, 20ng/ml bFGF, 20ng/ml bFGF EGF and 1mg/ml BSA (Gemini, 700-104P). After 2-3 days, CNCCs were split 1:3 and the following day cells were fed with neural crest long-term media. Long term media: neural crest maintenance media plus 50pg/ml BMP2 (PeproTech, 120-02) and 3uM CHIR-99021 (Selleck Chemicals, S2924). CNCCs were then passaged twice to passage 4 and processed for various assays.

#### cDNA preparation and reverse transcriptase digital droplet PCR (RT-ddPCR)

Total RNA was extracted from late (passage 4, P4) CNCCs differentiated from hESC wild-type or mutant cell-lines using Trizol reagent (Invitrogen) for at least two differentiations. 500ng – 1ug RNA was used to generate cDNA using the SuperScript Vilo IV MasterMix with ezDNase enzyme (Invitrogen, 11766050). Primers and locked nucleic acid (LNA) probes were designed by the IDT custom design service to the human SOX9 3’UTR, centered on the rs74999341 T/C SNP – a HEX LNA probe detects the T-allele and a FAM LNA probe detects the C-allele (Table S3). cDNA dilution factor was determined using qPCR with 1X PrimeTime Gene Expression Master Mix (IDT), 500 nM primers and 250 nM probes, run on LightCycler 480 (Roche). ddPCR reactions were performed using diluted cDNA (10-100X diluted), 900 nM primers and 250 nM probes and 1X ddPCR Supermix for probes (no dUTP, BioRad). ddPCR droplets were generated using the QX200 Droplet Generator (BioRad) and droplets were read using QX200 Droplet Reader (BioRad) and analyzed using the QuantaSoft Software (BioRad).

#### Chromatin immunoprecipitation digital droplet PCR (ChIP-ddPCR), ChIP-qPCR or ChIP-seq

5-15 million cells were cross-linked per ChIP experiment in 2mL PBS per 6-well with 1% methanol-free formaldehyde for 5-10 min and quenched with a final concentration of 0.125M glycine for 5 min with nutation. Cross-linked cells were washed with PBS, scraped and pelleted by centrifugation, flash-frozen in liquid nitrogen and stored at -80°C. Samples were defrosted on ice and resuspended in 5mL LB1 (50 mM HEPES-KOH pH 7.5, 140 mM NaCl, 1 mM EDTA, 10% glycerol, 0.5% NP-40, 0.25% Triton X-100, with 1X cOmplete Protease Inhibitor Cocktail and 1 mM PMSF) and rotated vertically for 10 min at 4°C. Samples were centrifuged for 5 min at 1350 x g at 4°C, and resuspended in 5mL LB2 (10 mM Tris, 200 mM NaCl, 1 mM EDTA, 0.5 mM EGTA, with 1X cOmplete Protease Inhibitor Cocktail and 1mM PMSF) and rotated vertically for 10 min at 4°C. Samples were centrifuged for 5 min at 1350 x g at 4°C, and resuspended in 1mL LB3 per 10 million cells (up to 1 mL per ChIP). Samples were sonicated in 1mL AFA tubes for 5 min on E220 evolution Covaris with settings Peak power = 140, Duty Factor = 10, Cycles per burst = 200 to achieve chromatin sized approximately 500-2000bp. Following sonication, samples were re-combined (if aliquoted for sonication), Triton X-100 was added to the fragmented chromatin to a final concentration of 1%, and the chromatin divided for input (1%–2%) and ChIP samples. 5 ug anti-histone H3K27ac (Active Motif, 39133) antibody,10 μL anti-CTCF (Cell Signaling, 2899S) and 7.5 ug anti-RAD21 (Abcam, ab992) antibody was added per ChIP sample, and incubated overnight at 4°C with rotation. Protein G Dynabeads (ThermoFisher) were first blocked with Block solution (0.5% BSA (w/v) in 1X PBS) and then added to cleared chromatin to bind antibody-bound chromatin during a 4-6 hour incubation. Chromatin-bound Dynabeads were washed at least 6 times with chilled RIPA wash buffer (50 mM HEPES-KOH pH 7.5, 500 mM LiCl, 1 mM EDTA, 1% NP-40, 0.7% Na-Deoxycholate), followed by a wash with chilled TE + 50 mM NaCl. Chromatin was eluted for 15-30 min in Elution Buffer (50 mM Tris, 10 mM EDTA, 1% SDS) at 65°C with frequent vortexing. The ChIP and input samples were then incubated at 65°C overnight to reverse cross-links (12-16 hours). Samples were diluted and sequentially digested with RNase A (0.2 mg/mL) for 2 hours at 37°C followed by Proteinase K (0.2 mg/mL) for 2 hours at 55°C to digest protein. ChIP and input samples were purified by phenol-chloroform-isoamylalcohol extraction and precipitation with final concentration 70% ethanol, 0.3M NaOAc pH 5.2 and 1.5 mL glycogen.

For ChIP-ddPCR, primers were designed to amplify across the EC1.45, E1.35 and EC1.25 regions, and allele-specific probes were designed to distinguish between wild-type and mutated alleles (Table S3). ChIP and input dilution factors were determined using qPCR with 1X PrimeTime Gene Expression Master Mix (IDT), 500 nM primers and 250 nM probes, run on LightCycler 480 (Roche). ddPCR reactions were performed using ChIP DNA (40X diluted) and input DNA (640X diluted), 900 nM primers and 250 nM probes and 1X ddPCR Supermix for probes (no dUTP, BioRad). ddPCR droplets were generated using the QX200 Droplet Generator (BioRad) and droplets read using QX200 Droplet Reader (BioRad) and analyzed using the QuantaSoft Software (BioRad).

For ChIP-qPCR, primers were designed to amplify adjacent to the SSE1.35 and SSEProm CTCF site (Table S3). ChIP DNA and input DNA were diluted 5X and amplified using 500 nM primers and SensiFAST SYBR (No-ROX Kit, Bioline) using a LightCycler 480 (Roche).

For ChIP-Seq library preparation, samples were quantified by Qubit dsDNA HS assay kit, and 30 ng of ChIP DNA was used for library preparation with end repair, A-tailing, and adaptor ligation (NEB). Following USER enzyme treatment, libraries were cleaned up with one round of single-side AMPure XP bead clean-up. Libraries were then amplified to add indices using NEBNext HiFi 2X PCR mix and NEBNext Multiplex Oligos for Illumina kit (NEB, E7335S) with 9 cycles (as determined by qPCR. ChIP libraries were purified by two rounds of double-sided AMPure XP bead clean-up to deplete adaptors. Library concentration and quality within ChIP or input groups was assessed by Qubit dsDNA HS assay kit and used to pool within ChIP or input groups. Pooled libraries were sequenced using Novaseq 6000 platform (2x 150bp). ChIP-seq raw reads were aligned to the human genome (hg19) using the bowtie2 (version 2.4.1). bigWig files for visualization were generated using deeptools v3.5.0 with the normalization setting : -- normalizeUsing RPGC.

#### ORCA

The *SOX9* ORCA probeswere designed as previously described.^10^^,^^77^ We designed ORCA probes to label 80 5kb-regions at 50kb intervals across a 4 Mb genomic region encompassing the *SOX9* regulatory domain and flanking TADs (Table S1 and S2).

#### Sample preparation

ORCA imaging experiments were performed following the protocol described.^10^^,^^77^ Briefly, hESCs and CNCCs were fixed in 4% PFA in 1x PBS for 10 min. Fixed cells were plated on a poly-D-lysine-coated 40 mm coverglass and permeabilized with 0.5% Triton-X in 1x PBS for 10 min, followed by 2 washes with 1x PBS. Cells were incubated for 5 minutes in 0.1 M HCl, followed by 3 washes with 1x PBS and 3 washes with 2x SSC. We then treated cells for 35 min in 2x SSC + 50% vol/vol formamide and 0.1% Tween. 3 μg SOX9 ORCA probe in 30 μl Hybridization buffer (2x SSC, 50% vol/vol formamide and 0.1% Tween) were added onto cells. Cells were then denatured for 3min at 90°C and incubated overnight at 42°C. After the hybridization, cells were washed twice for 10min in 2x SSC at 42°C, then postfixed in 2% GA, 8% PFA in 1x PBS for 30 min. Cells were then washed in 2x SSC and either imaged directly or stored for up to a week at 4°C.

#### Image acquisition

Samples were imaged on the custom microscopy and microfluidics setup as described.^10^^,^^77^ Briefly, samples were mounted into a flow chamber. Readout barcodes were visualized sequentially by complementary oligos carrying Cy5 dye followed strand displacement and washes. A Cy3 oligo that label all barcodes were also imaged at each round of imaging as fiducial spots. The fiducial spots were subsequently used for spot calling and registration in the image analysis pipeline.

#### Image processing

Spot calling, localization, and drift correction were performed as previously described.^10^^,^^77^ Software for processing the raw data is available at https://doi.org/10.5281/zenodo.7698979.

#### Polymer simulation

Polymer simulations were conducted using the open source polychrom software^52^ from open2c, which builds on previously published approaches for simulating chromatin structure and loop extrusion.^20^^,^^78^ This software constructs Langevin dynamic simulations of flexible polymers moving under thermal noise with user defined energy potentials to describe the bending stiffness, bond stiffness, and molecular interactions among monomers, including the links produced by loop extrusion factors. The simulations use the freely distributed openMM framework,^79^ widely used in molecular dynamics and protein structure simulations, to provide GPU accelerated computation. To simulate the “reel-in” model of stripe formation, we added targeted loading to this framework. This expanded version of the software is available at https://doi.org/10.5281/zenodo.7698987. Annotated, executable scripts with the parameters used to simulate both the “reel-in” and the “multi-loop” models will be provided at https://doi.org/10.5281/zenodo.7698897.

Briefly, both the “reel-in” and the “multi-loop” simulations used a polymer chain consisting of 1000 monomers, with extrusion blocking elements (simulating the CTCF structural elements) positioned internally (at monomer 200 and 800). In the “reel-in” model, cohesin was primarily loaded at monomer 205 and 795. To explore the relative difference in loading strength, each cohesin loaded had a 10% probability of loading at monomer 205 and 90% probability of loading at monomer 795. In the “multi-loop” model cohesin loaded randomly throughout the domain instead. In the “reel-in” model, no more than two LEFs/cohesins were extruding at any given time, and LEFs/cohesins remained bound for an average of 800 monomer steps. In the “multi-loop” model up to 20 LEFs/coheins could be randomly loaded across the domain, but remained on the polymer long enough on average to traverse only 40 monomer steps. Capture probability for an extrusion blocking element to stop a LEF/cohesin, average residence time of a LEF/cohesin at the capture site, and polymer mechanical properties were otherwise identical between both simulations. Further details can be seen in deposited scripts.

### Quantification and statistical analysis

#### ORCA image analysis

After spot calling and localization, barcodes with low hybridization efficiency (< 10%) were filtered. Traces with low number of barcodes labeled (< 30%) and very high number of barcodes labeled (> 90%) were then filtered. Pairwise distances were calculated for all filtered barcodes and traces. Contact frequency is defined as the percentage of pairwise inter-probe distances below 250 nm. To estimate the confidence interval for the difference in contact frequency between conditions, we performed bootstrapping by randomly sampling with replacement from the data.

To estimate the volume of the chromatin domain, we calculated the Radius of gyration of each trace. **Radius of gyration (R_g_)** of the chromatin domain was computed from the vector data of molecule locations as ${R_{g}}^{2}=\frac{1}{N}\;\sum_{i=1}^{N}\;$(r_i_ – r_c_)^2^Where r_i_ is a vector of the coordinate of an individual localization and r_c_ is the centroid of all N localizations.

To estimate the average 3D structure of domain, we performed **non-metric multidimensional scaling (MDS)** on the average pairwise distance matrix from ORCA. We calculated Euclidean distances for all pairs of points in 3D using Kruskal's normalized stress criterion.

##### K-means clustering and tSNE

We used the pairwise distance from the points in *SOX9* TAD to *SOX9* gene (barcodes 11-52 to barcode 42). We only used traces that were labeled by more than 60% barcodes and interpolated the missing data points in these traces using the mean pairwise distance from the neighboring two barcodes. K-means clustering with different number of clusters was performed on the interpolated data and the optimal number of clusters was determined by the Elbow method. Clusters from K-means clustering were superimposed on the tSNE plots.

#### Analyzing and plotting ddPCR data

Concentration of the two alleles of *SOX9* was determined by RT-ddPCR using allele-specific HEX/FAM probes from the QuantaSoft Software. To plot allelic skew, concentration for the mutated allele was divided by the concentration for the wild-type allele and plotted as a ratio (red boxplots). For matched wild-type cells, the same ratio was calculated, and then the values were normalized such that the wildtype median equals to 1.

## Data Availability

• ChIP-seq data have been deposited at GEO. All probe coordinates and imaging data tables have been deposited on the 4DN public access server. External HiC data was obtained from the 4DN consortium. All data are publicly available as of the date of publication. Accession numbers are listed in the key resources table. • All original code has been deposited at Github and Zenodo and is publicly available as of the date of publication. DOIs are listed in the key resources table. • Any additional information required to reanalyze the data reported in this paper is available from the lead contact upon request.

## Supplemental information

Document S1. Figures S1–S6

Table S1. SOX9 ORAC probe: Sequences, related to STAR Methods

Table S2. SOX9 ORCA probe: Genomic coordinates, related to STAR Methods

Table S3. A list of primers and probes used for ChIP-ddPCR, RT-ddPCR, and qRT-PCR analyses, related to STAR Methods

Methods S1. GenBank files of sgRNAs and targeting constructs used for generating ΔE1.35/+ H9 cell lines, related to STAR Methods

Document S2. Article plus supplemental information
