# Supplementary material for: Structural elements promote architectural stripe formation and facilitate ultra-long-range gene regulation at a human disease locus
Source: Mol Cell. 2023 May 4;83(9):1446–1461.e6. doi: 10.1016/j.molcel.2023.03.009 (PMC13507663; doi:10.1016/j.molcel.2023.03.009)
Supplement: Supplementary file 1 [file mmc1.pdf]

Molecular Cell, Volume *83*

Supplemental information

Structural elements promote architectural stripe formation and facilitate ultra-long-range gene regulation at a human disease locus

Liang-Fu Chen, Hannah Katherine Long, Minhee Park, Tomek Swigut, Alistair Nicol Boettiger, and Joanna Wysocka

Supplementary Figure 1. Related to Figure 1

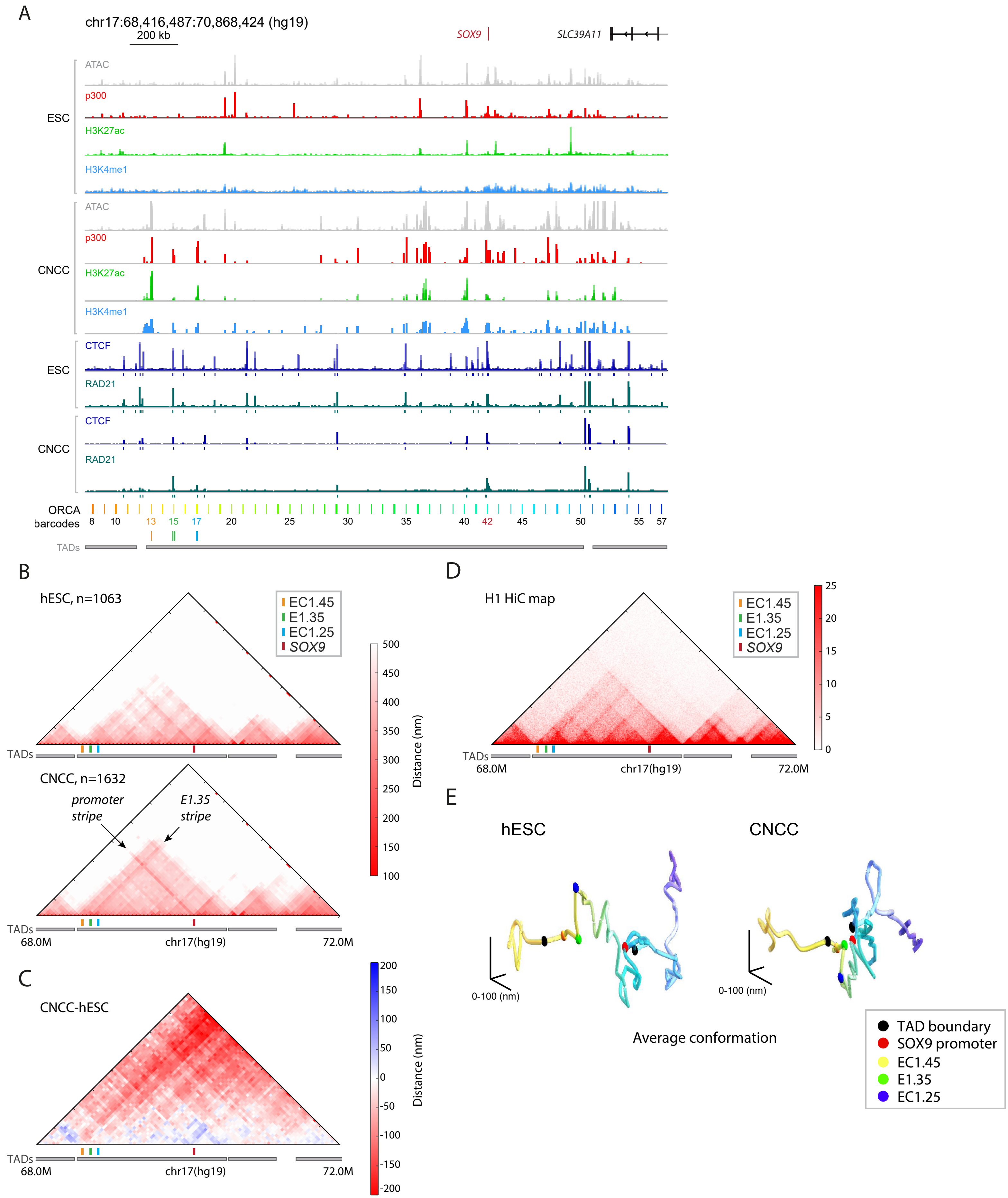

Supplementary Figure 2. Related to Figure 2

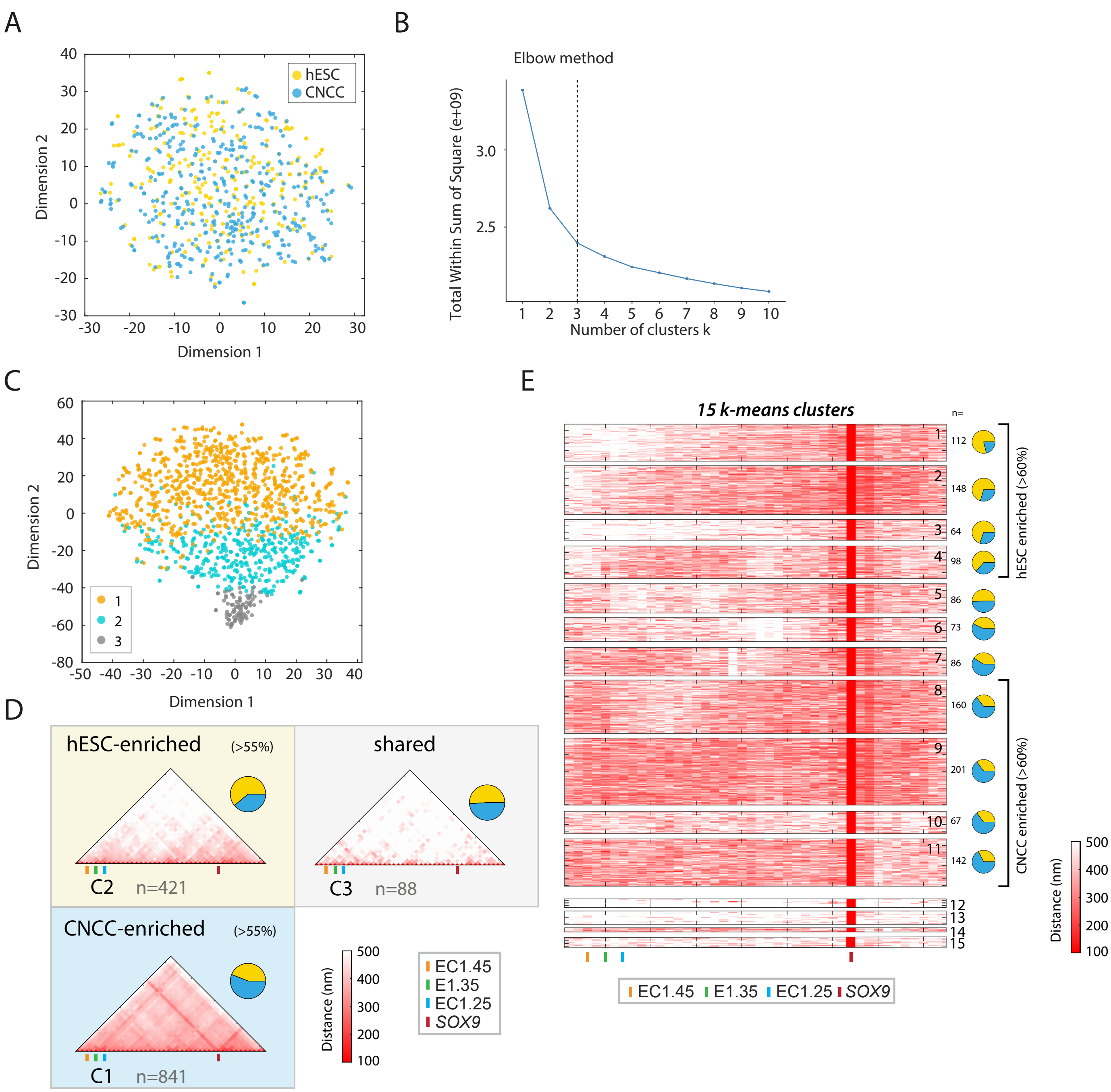

Supplementary Figure 3. Related to Figure 3

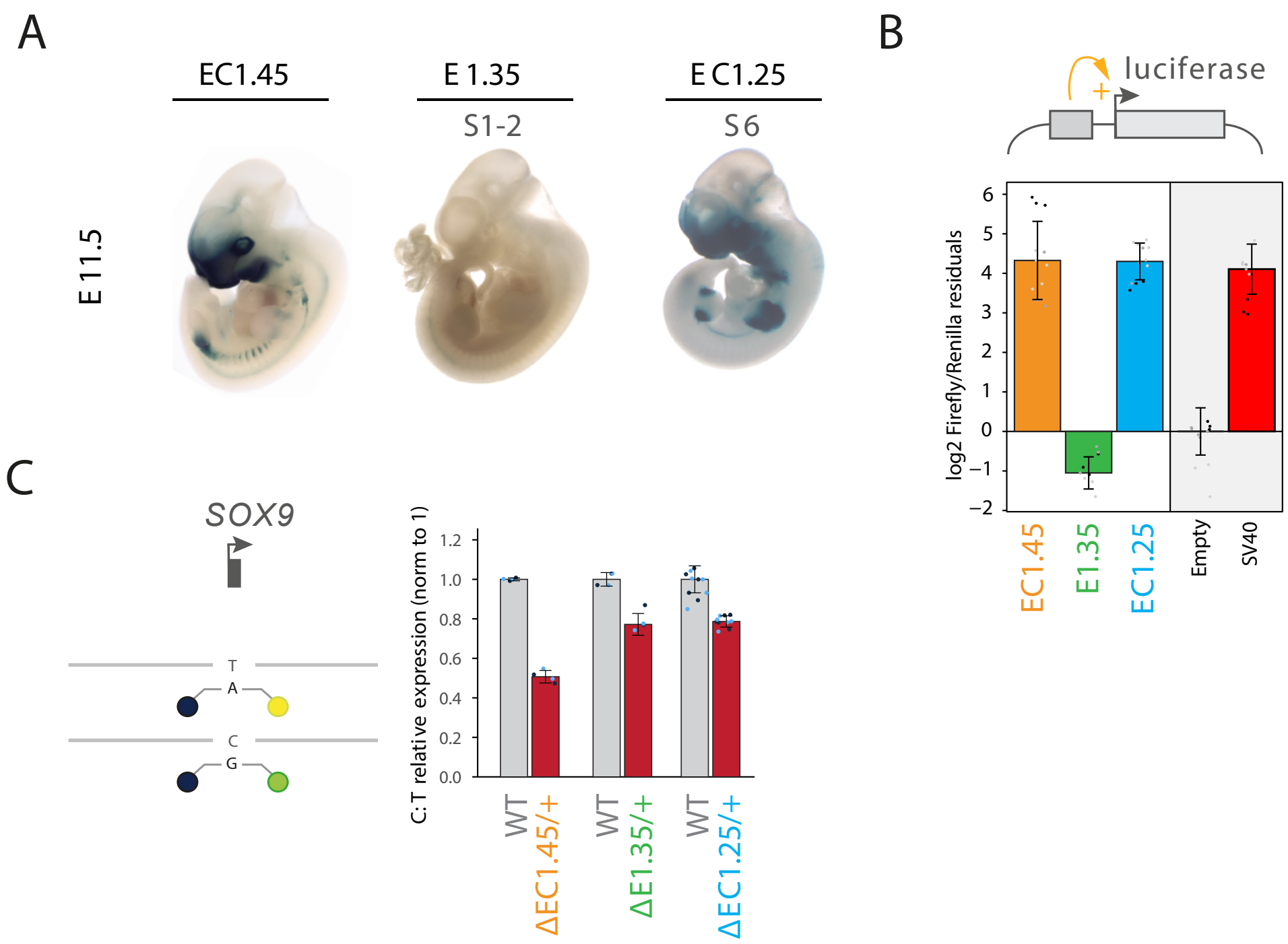

Supplementary Figure 4. Related to Figure 4

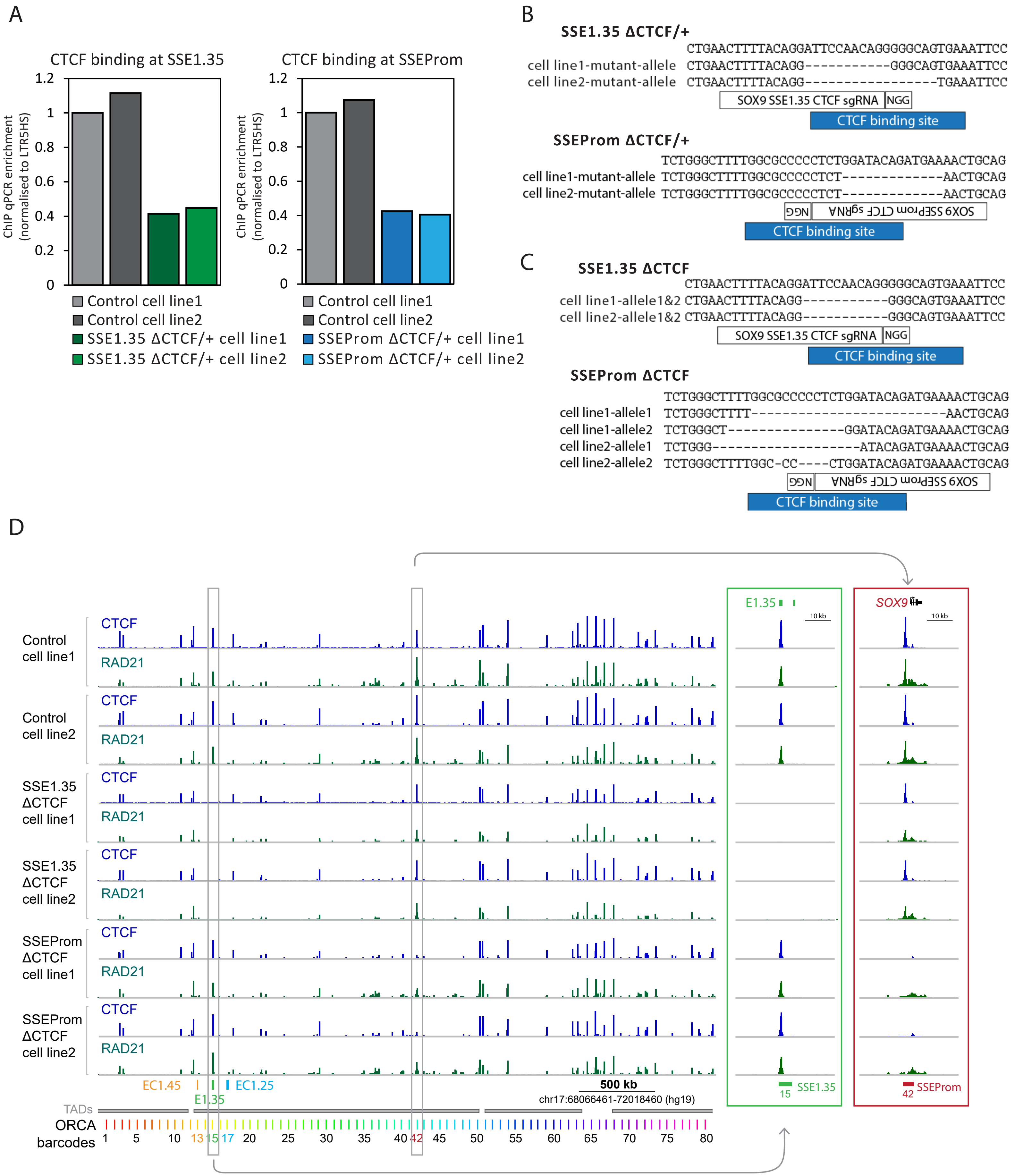

Supplementary Figure 5. Related to Figure 4

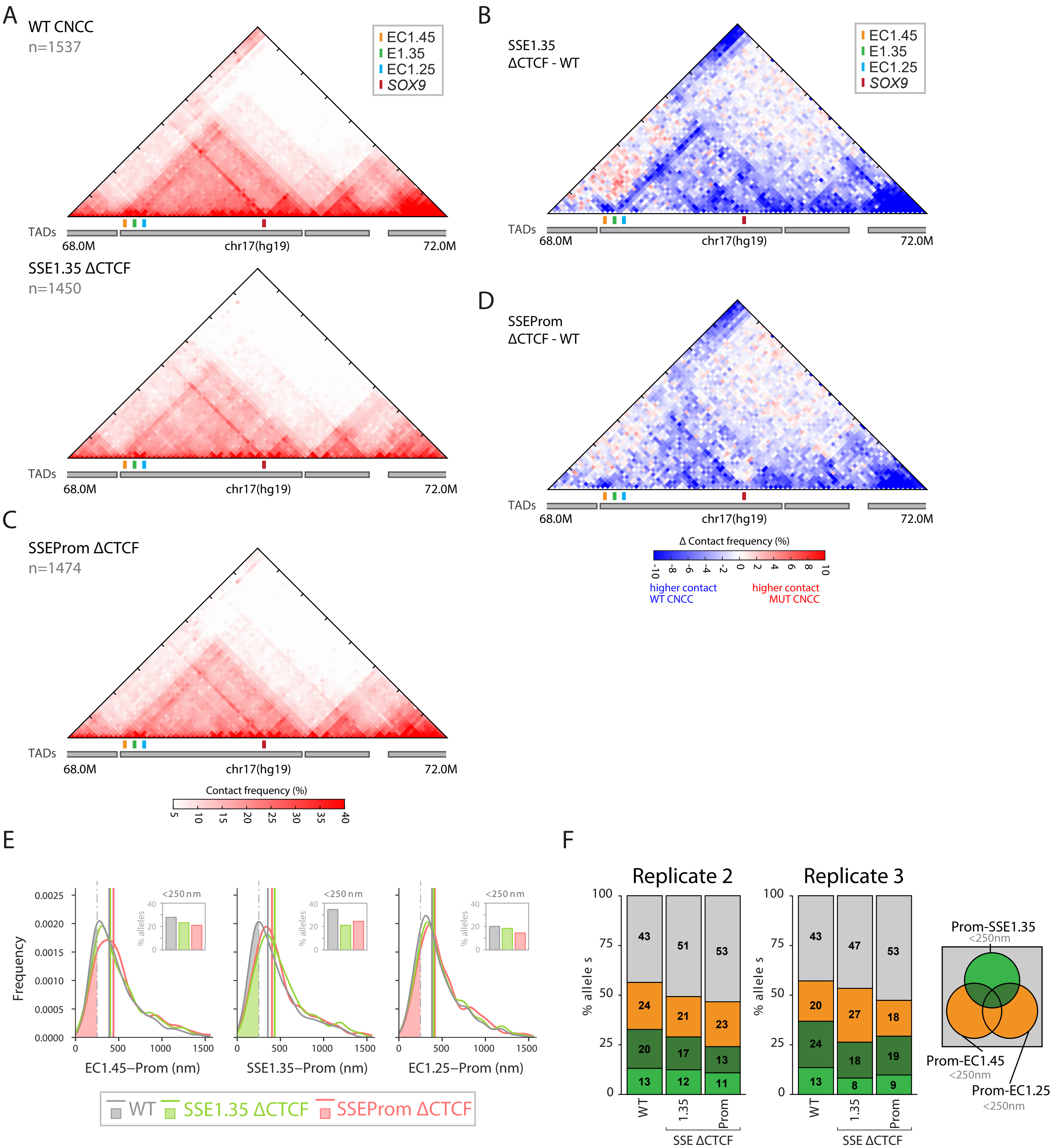

Supplementary Figure 6. Related to Figure 5

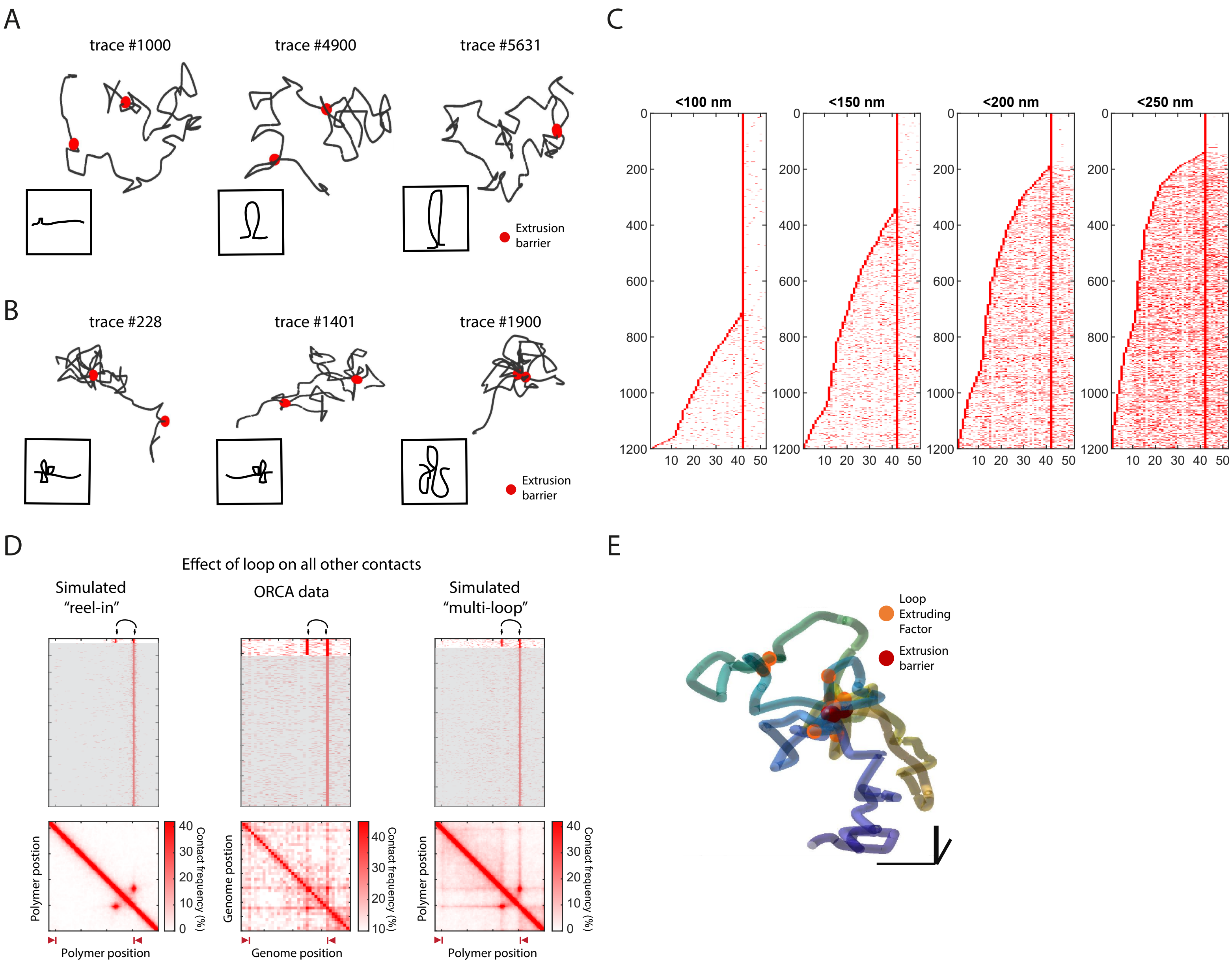

Supplementary Figure 1. ChIP-seq, ORCA distance maps and the average 3D structure of the *SOX9* locus from hESCs and CNCCs. Related to Figure1.

A) ATAC-seq and p300, H3K27ac, H3K4me1, CTCF and cohesin ChIP-seq data from hESCs and CNCCs for the locus surrounding the *SOX9* gene. Enhancer clusters EC1.45 and EC1.25 are highlighted along with the nearby E1.35 element. ORCA barcodes are annotated across the domain, along with TADs. B) Absolute pairwise distance maps for all ORCA barcodes across the *SOX9* locus from ORCA imaging data for hESC (upper) and CNCCs (lower). C) Absolute pairwise distance subtraction maps for CNCC-hESC. D) Hi-C contact frequency maps for H1 hESCs. E) MDS plots of the average 3D structure of the *SOX9* locus and flanking regions in hESCs (left) and CNCCs (right). Barcodes are highlighted that overlap interesting genomic features. Data from Figure 1B.

Supplementary Figure 2. K-means clustering with 3 clusters and pairwise distances of the *SOX9* promoter to all other regions in the surrounding domain in the 15 K-means clusters. Related to Figure 2.

A) A tSNE plot representing all pairwise distances of all versus all positions across the *SOX9* TAD (data from Figure 1). B) Elbow method to determine optimal number of K-means clusters for all pairwise distances of the *SOX9* promoter to the remainder of the TAD (data from Figure 1). C) tSNE plot from Figure 2A with 3 clusters from K-means clustering indicated. D) Contact frequency maps from all traces for each cluster from C. E) Heatmaps representing absolute pairwise distances of the *SOX9* promoter with all other regions in the surrounding domain, showing all traces from each of the 15 K-means clusters from Figure 2B.

Supplementary Figure 3. The PRS locus E1.35 element regulates *SOX9* expression in the absence of enhancer activity. Related to Figure 3.

A) LacZ reporter assay for EC1.45, E1.35 and EC1.25 at embryonic day E11.5. B) Luciferase assay for EC1.45, E1.35 and EC1.25 in CNCCs. C) Allelic expression of *SOX9* for wildtype and heterozygous EC1.45, E1.35 and EC1.25 mutant CNCCs, plotting mutant versus wildtype allelic expression measured by RT-ddPCR, with wildtype expression normalized to 1. All data (apart from E1.35 deletion expression data) from (**Long et al.**, **2020**)[36].

Supplementary Figure 4. Validation of SSE CTCF mutant clones. Related to Figure 4.

A) ChIP-qPCR for CTCF at SSE1.35 for two heterozygous mutant clones (E1.35 ΔCTCF/+), compared to wildtype (left), and SSEProm for heterozygous mutant clones (SSEProm ΔCTCF/+) compared to wildtype (right). B) Multiple sequence alignment illustrating the mutation allele for the heterozygous CTCF mutant clones. C) Multiple sequence alignment illustrating the indel mutations for each of the alleles for the homozygous CTCF mutant clones. D) CTCF and RAD21 ChIP-seq for wildtype, E1.35 ΔCTCF and SSEProm ΔCTCF CNCCs across *SOX9* TAD. ORCA barcodes 1-80 indicated chr17:68066461-72018460 (hg19).

Supplementary Figure 5. ORCA contact frequency maps across *SOX9* and neighboring TADs and two additional replicates for multiway interactions analysis. Related to Figure 4.

A) ORCA contact frequency maps across *SOX9* and neighboring TADs for pairwise distances <250 nm for wild-type (upper) and E1.35 CTCF deletion (SSE1.35 ΔCTCF) CNCCs (lower). Data from Figure 4C. B) Contact frequency subtraction maps for E1.35 CTCF deletion (SSE1.35 ΔCTCF) compared to wildtype. C) As A, for SSEProm CTCF deletion CNCCs (SSEProm ΔCTCF). Data from Figure 4E. D) As B, for SSEProm ΔCTCF CNCCs. E) Frequency plots showing pairwise distance distributions for wild-type, SSE1.35 ΔCTCF and SSEProm ΔCTCF CNCCs for EC1.45, E1.35 (SSE1.35) and EC1.25 to the *SOX9* promoter from left to right. Percentage of alleles with respective pairwise distances <250 nm are shown as an inset. F) Two additional replicates for Figure 4G. Percentage of alleles with promoter proximity to EC1.45, SSE1.35 and/or EC1.25 including multiway interactions for wild-type, SSE1.35 ΔCTCF and SSEProm ΔCTCF CNCCs.

Supplementary Figure 6. Exemplar traces from polymer simulations and additional example of pairwise contacts from one stripe anchor to a specific point in the domain. Related to Figure 5.

A) Exemplar traces, #1000, #1900 and #5631 from the “reel-in” model. B) Exemplar traces, #228, #1401 and #1900 from the “multi-loop” model. C) Single chromatin ORCA traces of contacts to the stripe anchor at the right side of the domain at different threshold for contacts as indicated above each plot. D) As Figure 5D. Heatmap of iterations (polymer simulations) or traces (ORCA imaging) for pairwise contacts from one stripe anchor across the domain ordered by interactions with a specific point (black arrows) in the domain (upper). Population-level contact frequency maps from traces showing contacts in the upper traces (lower). E) An exemplar trace from the “multi-loop” model showing multiple loop conformation and central positioning of the stripe anchors.
